# Effectiveness of tofacitinib versus tumor necrosis factor inhibitors and in those receiving tofacitinib as different lines of therapy in patients with rheumatoid arthritis: results from the United States CorEvitas Rheumatoid Arthritis Registry

**DOI:** 10.1007/s10067-024-07245-3

**Published:** 2024-12-20

**Authors:** Leslie R. Harrold, Clifton O. Bingham, Janet E. Pope, Jacqueline O’Brien, Page C. Moore, Carla Roberts-Toler, Miao Yu, Lindsay L. Sweet, Ahmed Shelbaya, Karim R. Masri

**Affiliations:** 1 CorEvitas, LLC, Waltham, MA USA; 2 Department of Medicine, Division of Rheumatology, University of Massachusetts Chan Medical School, Worcester, MA USA; 3 Division of Rheumatology, Department of Medicine, Johns Hopkins University School of Medicine, Baltimore, MD USA; 4 Medicine, Division Rheumatology, Western University, London, ON Canada; 5 Pfizer Inc, Collegeville, PA USA; 6 Pfizer Inc, 66 Hudson Boulevard, New York, NY 10001 USA

**Keywords:** Comparative effectiveness, Disease-modifying antirheumatic drugs (DMARDs), Registry, Rheumatoid arthritis

## Abstract

**Objectives:**

To compare effectiveness of tofacitinib versus tumor necrosis factor inhibitors (TNFi), and across tofacitinib lines of therapy, in patients with rheumatoid arthritis (RA), using US CorEvitas RA Registry data.

**Methods:**

Analysis included patients with RA initiating tofacitinib or TNFi with a 12-month follow-up visit between November 2012–February 2021. Primary (Clinical Disease Activity Index-defined low disease activity [CDAI-LDA: CDAI ≤ 10]) and secondary (clinical/disease activity/patient-reported) effectiveness outcomes were assessed at month 12. Outcomes were stratified by treatment regimen (overall tofacitinib vs overall TNFi/tofacitinib monotherapy vs tofacitinib combination therapy/TNFi monotherapy vs TNFi combination therapy/tofacitinib monotherapy vs TNFi combination therapy/tofacitinib combination therapy vs TNFi combination therapy), or tofacitinib line of therapy (2nd/3rd/ ≥ 4th line).

**Results:**

3,481 eligible patients initiated tofacitinib (*n* = 805) or TNFi (*n* = 2,676). Improvements in effectiveness at month 12 were generally similar across treatment regimens; 25.1% and 30.1% of overall tofacitinib and TNFi initiators achieved CDAI-LDA, respectively (odds ratio 1.29 [95% confidence interval (CI) 0.94, 1.76]). Odds ratios (95% CIs) for achieving CDAI-LDA at 12 months were 0.70 (0.36, 1.37) for 3rd- versus 2nd-line, and 1.09 (0.63, 1.88) for 3rd- versus ≥ 4th-line tofacitinib initiators. At month 12, mean change from baseline in CDAI was greater among 3rd- versus ≥ 4th-line tofacitinib initiators, and mean Health Assessment Questionnaire and patient-reported pain were greater in 3rd- versus 2nd-line and ≥ 4th- versus 3rd-line tofacitinib initiators.

**Conclusions:**

Generally, there were no differences in effectiveness between tofacitinib versus TNFi regimens. Few differences were observed between tofacitinib lines of therapy; sample sizes were small for 2nd/3rd-line initiators.

**Study registration:**

NCT01402661 (ClinicalTrials.gov; July 25, 2011).

**Table:** 

**Key Points** • *Using data from the US CorEvitas rheumatoid arthritis (RA) Registry, this study compared the effectiveness of tofacitinib versus tumor necrosis factor inhibitors (TNFi) and across tofacitinib lines of therapy.* • *Effectiveness of tofacitinib was similar to TNFi regimens up to month 12, while differences in some effectiveness outcomes at month 12 were observed with tofacitinib across different lines of therapy.* • *The findings of this study may inform future treatment decision-making in patients with RA.*

**Supplementary Information:**

The online version contains supplementary material available at 10.1007/s10067-024-07245-3.

## Introduction

Rheumatoid arthritis (RA) is a chronic, systemic autoimmune disease characterized by stiffness, swollen joints, and pain [1], which affects between 0.41–0.54% of insured adults in the United States (US) and 0.46% of adults worldwide [2, 3]. Early diagnosis and treatment of RA are crucial components of a treat-to-target approach that aims to reduce or prevent progression of joint damage and optimize physical functioning [1, 4].

Patients with RA typically initiate 1st-line treatment with conventional synthetic disease-modifying antirheumatic drugs (csDMARDs), such as methotrexate, as recommended by the American College of Rheumatology (ACR) and the European Alliance of Associations for Rheumatology (EULAR) [5, 6]. In patients who do not adequately respond, or are intolerant to methotrexate, ACR and EULAR guidelines recommend initiating 2nd-line treatment including either a biologic (b)DMARD, such as a tumor necrosis factor inhibitor (TNFi), or a targeted synthetic DMARD, such as a Janus kinase (JAK) inhibitor, either alone or in combination with a csDMARD [5, 6]. According to EULAR guidelines, certain risk factors should be considered when using JAK inhibitors [5]. RA treatment is often based on a ‘trial-and-error’ approach; therefore, more comparative studies are needed to improve treatment decisions for individual patients [7].

The efficacy and safety of the oral JAK inhibitor tofacitinib in patients with RA have been demonstrated in Phase 3 and Phase 3b/4 randomized controlled trials [8–15] and in long-term extension studies [16–18]. ORAL Surveillance, a post-authorization safety study of tofacitinib in cardiovascular risk-enriched patients with RA, showed that rates of major adverse cardiovascular events and cancers were higher with tofacitinib versus TNFi [19]; this resulted in a US Food and Drug Administration label change to indicate tofacitinib for patients with RA who have had an inadequate response or intolerance to ≥ 1 TNFi [20]. This change has not yet been reflected in the current ACR treatment guidelines [6].

Real-world studies complement the findings of randomized controlled trials by providing evidence on the safety and efficacy of a medical product resulting from routine clinical care settings [21]. Previous analyses of real-world registries and claims databases of patients with RA have shown that tofacitinib is initiated at later lines of therapy and more frequently as monotherapy than TNFi [22–26], and that effectiveness is generally similar with tofacitinib versus TNFi [23–26]. A study by Reed et al. using data from 2012 to 2016 from the US CorEvitas RA Registry (formerly known as Corrona) demonstrated that in the 3rd or 4th line of therapy, tofacitinib administered with or without methotrexate achieved similar effectiveness to TNFi with methotrexate at 6-month follow-up [23]; due to low patient numbers, effectiveness could not be meaningfully assessed in those initiating tofacitinib as a 2nd line of therapy.

Here, we expand on the study by Reed and colleagues [23] by providing an updated analysis (observation period: November 2012 to February 2021) of tofacitinib use in patients with RA enrolled in the US CorEvitas RA Registry, with a focus on 12-month follow-up. Specifically, we sought to compare effectiveness outcomes in: 1) those initiating tofacitinib versus TNFi administered as monotherapy or in combination with csDMARDs; and 2) those initiating tofacitinib as different lines of therapy (2nd, 3rd, or ≥ 4th line).

## Materials and methods

### Data source

The US CorEvitas RA Registry (NCT01402661), established in 2001 and described previously [27, 28], is an independent, prospective, national observational cohort of patients with RA, and is the largest ongoing US registry for RA. The registry collects data on patient demographics, lifestyle factors, RA disease characteristics, comorbidities, medication history, clinical outcomes, and patient-reported outcomes from patients and their treating healthcare providers at regular visits. As of March 31, 2021, the registry included data from 203 active private and academic clinical sites with 871 physicians across 42 states in the US; over 56,000 patients with RA were enrolled, representing over 214,000 patient-years of data.

### Study population

This study included patients aged ≥ 18 years enrolled in the US CorEvitas RA Registry with rheumatologist-diagnosed RA, who initiated tofacitinib or a TNFi (adalimumab, etanercept, infliximab, golimumab, or certolizumab pegol), as monotherapy or combination therapy, on or after November 6, 2012 (US Food and Drug Administration approval date for tofacitinib), and who had a 12-month (primary analysis) or 6-month (secondary analysis) follow-up visit on or before February 28, 2021. A 12-month or 6-month visit was defined as a clinical visit to the rheumatologist 10–14 months or 4–8 months from the treatment initiation date, respectively. If the patient had > 1 visit between 10–14 months or 4–8 months, the visit closest to the 12- or 6-month time point was selected. Patients could be included in both the 12-month and 6-month analysis groups if they had eligible visits.

This study was performed in accordance with the Declaration of Helsinki and the Guidelines for Good Pharmacoepidemiology Practice. All participating investigators were required to obtain full institutional review board (IRB) approval for conducting non-interventional research involving human patients with a limited dataset. Sponsor approval and continuing review were obtained from the New England Independent Review Board (no. 120160610). For academic investigative sites that did not receive authorization to use the central IRB, full board approval was obtained from the respective governing IRBs, and documentation of approval was submitted to CorEvitas, LLC prior to the initiation of any study procedures. All patients in the registry were required to provide written informed consent and authorization prior to participating in the study.

### Outcomes

Outcomes were assessed in patients with a 12-month visit and/or 6-month visit.

Data collected at baseline included patient demographics and disease characteristics, comorbidities, RA treatment history and concomitant therapies, physician-reported disease activity measures, and patient-reported outcome measures (e.g., pain, fatigue, Health Assessment Questionnaire [HAQ], and morning stiffness).

The primary effectiveness outcome (binary outcome) was the proportion of patients achieving Clinical Disease Activity Index-defined low disease activity (CDAI-LDA: CDAI score of ≤ 10 among those with scores > 10 at baseline). Continuous secondary effectiveness outcomes included change from baseline in CDAI and patient-reported outcome scores (HAQ, patient pain [0–100 mm Visual Analog Scale (VAS)], patient fatigue [0–100 mm VAS], and morning stiffness [hours]). Binary secondary effectiveness outcomes were the proportions of patients achieving CDAI-defined remission (CDAI score of ≤ 2.8 among those with CDAI > 2.8 at baseline), modified ACR (mACR) 20/50/70 responses (which show high correlation with unmodified ACR responses [29]), Disease Activity Score in 28 joints, erythrocyte sedimentation rate (DAS28-4[ESR])-defined LDA/remission (DAS28-4[ESR] ≤ 3.2 among patients with DAS28-4[ESR] > 3.2 at baseline), HAQ decrease of ≥ 0.22 from baseline (minimum clinically important difference; calculated among patients with HAQ > 0.22 at baseline), and mild pain (≤ 20 mm VAS; calculated among patients with pain > 20 mm VAS at the baseline).

### Statistical analyses

Patients were stratified by treatment regimen or tofacitinib line of therapy.

The following treatment regimens were evaluated: 1) overall tofacitinib versus overall TNFi; 2) tofacitinib monotherapy versus tofacitinib combination therapy; 3) TNFi monotherapy versus TNFi combination therapy; 4) tofacitinib monotherapy versus TNFi combination therapy; and 5) tofacitinib combination therapy versus TNFi combination therapy. Monotherapy was defined as initiation of tofacitinib or TNFi without concomitant csDMARDs. Combination therapy was defined as initiation of tofacitinib or TNFi with concomitant csDMARDs (methotrexate, leflunomide, sulfasalazine, and hydroxychloroquine). Overall tofacitinib or overall TNFi included all patients receiving monotherapy or combination therapy within each treatment arm.

When evaluating patients by tofacitinib line of therapy, 2nd line was defined as prior use of methotrexate and no prior use of bDMARDs; 3rd line was defined as prior use of methotrexate and prior use of 1 bDMARD; and ≥ 4th line was defined as prior use of methotrexate and prior use of ≥ 2 bDMARDs.

Patient demographics and baseline characteristics across treatment regimens were summarized descriptively using unmatched and propensity score matched data. For propensity score matching, patients were first frequency matched by line of therapy; any remaining imbalance between the groups was assessed using standardized differences, with differences < 0.1 indicative of a negligible difference between treatment groups. A propensity score model was then used to match the comparison groups by age, sex, disease duration, baseline CDAI, and any covariates that were imbalanced (i.e., standardized mean difference > 0.1). Covariates were excluded from the model if their inclusion resulted in a decrease in sample size of > 10%.

For the analysis of treatment regimens, the primary and secondary effectiveness outcomes at months 12 and 6 were compared using propensity score matched data. Logistic regression models were then used to compare binary outcomes between treatment regimens, providing odds ratios (ORs) and 95% confidence intervals (CIs). Linear regression models were used to compare continuous outcomes between treatment regimens, providing mean differences and 95% CIs. For mean differences between TNFi and tofacitinib regimens, a negative value indicated a greater response or outcome with tofacitinib versus TNFi. Models were adjusted for covariates that remained imbalanced after propensity score matching.

For the tofacitinib line of therapy analysis, primary and secondary effectiveness outcomes at months 12 and 6 were compared using multivariable adjusted logistic (binary outcomes) or linear (continuous outcomes) regression models. Models were adjusted for tofacitinib exposure, age, duration of RA, race, sex, and baseline CDAI. When comparing 3rd-line treatment with 2nd-line and ≥ 4th-line treatment, binary effectiveness outcomes are presented as ORs with 95% CIs, and continuous outcomes are presented as mean differences with 95% CIs.

For patients who discontinued tofacitinib or a TNFi and did not switch to another bDMARD or JAK inhibitor within 12 or 6 months, the outcome value at 12 or 6 months was used for continuous variables. Patients who discontinued tofacitinib or a TNFi and switched to another bDMARD or JAK inhibitor within 12 or 6 months were considered non-responders in binary outcome analyses. In continuous outcome analyses, the value at the switch visit was used if available, or the value was set to missing if the patient did not have a switch visit between the initiation visit and 12-month or 6-month visit.

An additional analysis was performed to compare effectiveness in overall tofacitinib and overall TNFi initiators who continued these treatments to the 12- or 6-month visit; these patients were defined as tofacitinib and TNFi non-switchers.

Analyses were performed using STATA Version 15.1 (StataCorp LLC., Texas, USA).

## Results

### Patients

From a total of 20,551 initiators (tofacitinib: *n* = 2,904; TNFi: *n* = 17,647), 3,481 patients had a qualifying 12-month visit, with 805 initiating tofacitinib (monotherapy: *n* = 334; combination therapy: *n* = 471) and 2,676 initiating TNFi (monotherapy: *n* = 620; combination therapy: *n* = 2,056 [Online Resource, Fig. S1]). A total of 4,326 patients had a qualifying 6-month visit, with 989 initiating tofacitinib (monotherapy: *n* = 415; combination therapy: *n* = 574) and 3,337 initiating TNFi (monotherapy: *n* = 757; combination therapy: *n* = 2,580 [Online Resource, Fig. S1]).

In patients with a 12-month visit, demographics, clinical characteristics, and disease activity measures at baseline were generally similar for overall tofacitinib and overall TNFi initiators, with a few exceptions (Tables 1 and 2). For overall tofacitinib versus overall TNFi initiators in the unmatched population, disease duration was longer (14.1 years vs 9.6 years), and fewer patients were bDMARD-naïve (16.8% vs 50.1%) and were taking concomitant methotrexate (41.4% vs 64.3% (Table 1)). A higher proportion of overall tofacitinib initiators had received ≥ 3 prior bDMARDs versus overall TNFi initiators (43.6% vs 10.1%, respectively (Table 1)).

**Table 1:** Demographics and baseline characteristics of overall tofacitinib and overall TNFi initiators with a 12-month visit^a^ (unmatched population)

	Overall tofacitinib initiators^b^ (*N* = 805)	Overall TNFi initiators^b^ (*N* = 2,676)	Standardized difference
Age, mean (SD)	59.9 (11.9)	58.9 (12.6)	0.085
Female, *n* (%)	651 (81.1)	2,094 (78.3)	0.069
Race, *n* (%)			
White	697 (88.1)	2,199 (83.2)	0.140
Hispanic	53 (6.7)	192 (7.3)	0.022
Black	30 (3.8)	167 (6.3)	0.116
Asian	6 (0.8)	40 (1.5)	0.071
Other	5 (0.6)	44 (1.7)	0.097
Medical insurance, *n* (%)			
None	7 (0.9)	53 (2.0)	0.094
Medicaid	50 (6.2)	151 (5.6)	0.024
Medicare	331 (41.1)	985 (36.8)	0.088
Private	553 (68.7)	1,865 (69.7)	0.022
Current smoker, *n* (%)	155 (19.4)	475 (18.0)	0.036
BMI, kg/m^2^, mean (SD)	29.9 (7.4)	30.7 (7.3)	0.105
History of bDMARD use, *n* (%)			
bDMARD naïve	135 (16.8)	1,342 (50.1)	0.756
1 prior bDMARD	158 (19.6)	793 (29.6)	0.234
2 prior bDMARDs	161 (20.0)	270 (10.1)	0.280
≥ 3 prior bDMARDs	351 (43.6)	271 (10.1)	0.816
Prednisone use, *n* (%)	220 (27.3)	724 (27.1)	0.006
Methotrexate use, *n* (%)	333 (41.4)	1,722 (64.3)	0.473
History of comorbidities, *n* (%)			
Cardiovascular disease^c^	133 (16.5)	320 (12.0)	0.131
Hypertension	273 (33.9)	885 (33.1)	0.018
Malignancy^d^	67 (8.3)	155 (5.8)	0.099
RA-related characteristics			
Duration of RA, years, mean (SD)	14.1 (10.3)	9.6 (9.7)	0.451
CDAI			
Mean (SD)	18.5 (13.1)	19.4 (14.1)	0.068
Median (Q1, Q3)	16.0 (7.9, 27.0)	17.0 (8.5, 27.0)	
CDAI, *n* (%)			
Remission (CDAI ≤ 2.8)	63 (7.8)	235 (8.8)	0.035
LDA (2.8 < CDAI ≤ 10)	205 (25.5)	557 (20.8)	0.110
MoDA (10 < CDAI ≤ 22)	255 (31.7)	934 (34.9)	0.068
HDA (22 < CDAI)	282 (35.0)	950 (35.5)	0.010
Tender joint count (28), mean (SD)	6.4 (6.9)	6.8 (7.2)	0.046
Swollen joint count (28), mean (SD)	4.5 (4.8)	4.9 (5.4)	0.080
DAS28-4(ESR), mean (SD)	4.2 (1.6)	4.1 (1.6)	0.088
Physician Global Assessment, mean (SD)	30.4 (21.7)	33.3 (23.1)	0.130
Patient Global Assessment, mean (SD)	45.4 (27.0)	44.4 (27.2)	0.037
Patient pain assessment (0–100 mm VAS), mean (SD)	48.9 (28.7)	47.1 (28.7)	0.062
Patient fatigue assessment (0–100 mm VAS), mean (SD)	49.9 (30.4)	47.1 (29.7)	0.094
HAQ (0–3), mean (SD)	1.1 (0.7)	1.0 (0.7)	0.158
mHAQ, mean (SD)	0.6 (0.5)	0.5 (0.5)	0.124
Patients with morning stiffness, *n* (%)	689 (86.0)	2,260 (85.0)	0.028
Morning stiffness duration, *n* (%)^e^			
< 30 min	103 (14.9)	399 (17.7)	0.062
30–59 min	139 (20.2)	431 (19.1)	0.031
60–119 min	190 (27.6)	625 (27.7)	0.005
≥ 120 min	252 (36.6)	791 (35.0)	0.037

*N* for each specific outcome may vary

Data shown are not propensity score matched

^a^12-month visit occurred 10–14 months after the index date

^b^Initiators included all patients who initiated tofacitinib or TNFi, including non-switchers

^c^Included carotid artery disease, acute coronary syndrome, cardiac arrest, congestive heart failure, coronary artery disease, myocardial infarction, revascularization procedures (coronary artery bypass grafting, coronary artery stents, or percutaneous coronary intervention), stroke, transient ischemic attack, unstable angina, ventricular arrhythmia, and other cardiovascular diseases

^d^History of lung cancer, breast cancer, lymphoma, skin cancer (basal, melanoma and squamous), or other cancer

^e^In patients who reported morning stiffness

*bDMARD* biologic disease-modifying antirheumatic drug; *BMI* body mass index; *CDAI* clinical disease activity index; *DAS28-4(ESR)* disease activity score in 28 joints, erythrocyte sedimentation rate; *HAQ* health assessment questionnaire; *HDA* high disease activity; *LDA* low disease activity; *MoDA* moderate disease activity; *mHAQ* modified health assessment questionnaire; *N* total number of patients; *n* number of patients with outcome; *Q* quartile; *RA* rheumatoid arthritis; *SD* standard deviation; *TNFi* tumor necrosis factor inhibitor; *VAS* visual analog scale

**Table 2:** Demographics and baseline characteristics of overall tofacitinib and overall TNFi initiators with a 12-month visit^a^ (propensity score matched population)

	Overall tofacitinib initiators^b^ (*N* = 611)	Overall TNFi initiators^b^ (*N* = 611)	Standardized difference
Age, mean (SD)	60.4 (11.8)	61.7 (11.7)	0.115
Female, *n* (%)	497 (81.3)	505 (82.7)	0.034
Race, *n* (%)			
White	530 (87.9)	516 (85.3)	0.076
Hispanic	42 (7.0)	34 (5.6)	0.055
Black	22 (3.6)	32 (5.3)	0.079
Asian	-^c^	-^c^	0.087
Other	-^d^	-^c^	-
Medical insurance, *n* (%)			
None	7 (1.1)	8 (1.3)	0.015
Medicaid	35 (5.7)	34 (5.6)	0.007
Medicare	264 (43.2)	292 (47.8)	0.092
Private	418 (68.4)	404 (66.1)	0.049
Current smoker, *n* (%)	114 (18.7)	110 (18.0)	0.017
BMI, kg/m^2^, mean (SD)	30.0 (7.6)	30.4 (7.2)	0.048
History of bDMARD use, *n* (%)			
bDMARD naïve	122 (20.0)	122 (20.0)	-
1 prior bDMARD	147 (24.1)	147 (24.1)	-
2 prior bDMARDs	119 (19.5)	119 (19.5)	-
≥ 3 prior bDMARDs	223 (36.5)	223 (36.5)	-
Prednisone use, *n* (%)	161 (26.4)	182 (29.8)	0.077
Methotrexate use, *n* (%)	284 (46.5)	196 (32.1)	0.298
History of comorbidities, *n* (%)			
Cardiovascular disease^e^	98 (16.0)	96 (15.7)	0.009
Hypertension	202 (33.1)	217 (35.5)	0.052
Malignancy^f^	56 (9.2)	51 (8.3)	0.029
RA-related characteristics			
Duration of RA, years, mean (SD)	13.5 (10.0)	14.6 (11.0)	0.098
CDAI			
Mean (SD)	19.2 (13.5)	18.2 (13.2)	0.076
Median (Q1, Q3)	17.0 (8.5, 27.0)	16.0 (7.8, 25.0)	
CDAI, *n* (%)			
Remission (CDAI ≤ 2.8)	39 (6.4)	46 (7.5)	0.045
LDA (2.8 < CDAI ≤ 10)	153 (25.0)	148 (24.2)	0.019
MoDA (10 < CDAI ≤ 22)	192 (31.4)	220 (36.0)	0.097
HDA (22 < CDAI)	227 (37.2)	197 (32.2)	0.103
Tender joint count (28), mean (SD)	6.7 (7.0)	6.5 (7.2)	0.033
Swollen joint count (28), mean (SD)	4.8 (5.1)	4.1 (4.7)	0.139
DAS28-4(ESR), mean (SD)	4.3 (1.6)	4.0 (1.6)	0.157
Physician Global Assessment, mean (SD)	31.3 (22.0)	30.7 (22.4)	0.029
Patient Global Assessment, mean (SD)	45.3 (26.6)	44.9 (26.5)	0.013
Patient pain assessment (0–100 mm VAS), mean (SD)	48.7 (28.5)	48.4 (27.9)	0.011
Patient fatigue assessment (0–100 mm VAS), mean (SD)	49.5 (30.5)	49.2 (29.2)	0.011
HAQ (0–3), mean (SD)	1.1 (0.7)	1.1 (0.7)	0.038
mHAQ, mean (SD)	0.6 (0.5)	0.6 (0.5)	0.011
Patients with morning stiffness, *n* (%)	530 (87.2)	523 (86.3)	0.026
Morning stiffness duration, *n* (%)^g^			
< 30 min	82 (13.5)	88 (14.6)	0.031
30–59 min	108 (17.8)	101 (16.7)	0.028
60–119 min	148 (24.4)	141 (23.4)	0.024
≥ 120 min	187 (30.9)	190 (31.5)	0.014

*N* for each specific outcome may vary

Overall tofacitinib and overall TNFi initiators were propensity matched by sex, age, duration of RA, CDAI, work status, insurance, serum positivity, methotrexate use, HAQ, patient-reported pain, patient-reported fatigue, and smoking status

^a^12-month visit occurred 10–14 months after the index date

^b^Initiators included all patients who initiated tofacitinib or TNFi, including non-switchers

^c^Cell value ≥ 5 patients but suppressed to minimize risk of reidentification

^d^Cell values of < 5 patients have been suppressed to minimize risk of reidentification

^e^Included carotid artery disease, acute coronary syndrome, cardiac arrest, congestive heart failure, coronary artery disease, myocardial infarction, revascularization procedures (coronary artery bypass grafting, coronary artery stents, or percutaneous coronary intervention), stroke, transient ischemic attack, unstable angina, ventricular arrhythmia, and other cardiovascular diseases

^f^History of lung cancer, breast cancer, lymphoma, skin cancer (basal, melanoma, and squamous), or other cancer

^g^In patients who reported morning stiffness

*bDMARD* biologic disease-modifying antirheumatic drug; *BMI* body mass index; *CDAI* clinical disease activity index, *DAS28-4(ESR)* disease activity score in 28 joints, erythrocyte sedimentation rate; *HAQ* health assessment questionnaire; *HDA* high disease activity; *LDA* low disease activity; *MoDA* moderate disease activity; *mHAQ* modified health assessment questionnaire; *N* total number of patients; *n* number of patients with outcome; *Q* quartile; *RA* rheumatoid arthritis; *SD* standard deviation; *TNFi* tumor necrosis factor inhibitor; *VAS* visual analog scale

Demographics and baseline characteristics for overall tofacitinib and overall TNFi initiators with a 6-month visit (Online Resource, Tables S1 and S2) were generally similar to those with a 12-month visit.

### Effectiveness outcomes stratified by treatment regimen

#### Overall tofacitinib versus overall TNFi

**Fig. 1:**
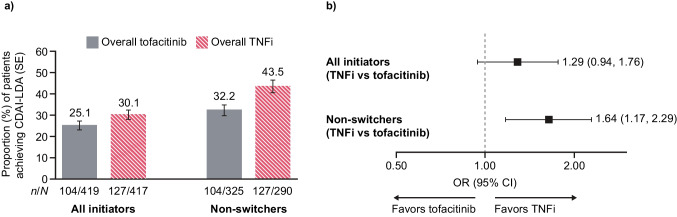
Rates of achieving CDAI-LDA (CDAI ≤ 10) for overall tofacitinib versus overall TNFi initiators (all initiators and non-switchers^a^) at month 12 in those with a month 12 visit^b^: **a** predicted probabilities and **b** ORs (95% CIs) (propensity score matched population). *n*/*N* denotes unadjusted response rates for patients with CDAI > 10 at baseline. Predicted probabilities and ORs are based on adjusted outcomes following propensity score matching. Covariates that remained imbalanced after propensity score matching and were included in the final model for the comparison of tofacitinib versus TNFi were baseline value and age, swollen joint count, and methotrexate use. ^a^Non-switchers were defined as patients who initiated tofacitinib or TNFi and continued these treatments to the 12- or 6-month visit.^b^12-month visit occurred 10–14 months after the index date. *CDAI* Clinical Disease Activity Index, *CI* confidence interval, *LDA* low disease activity, *N* total number of patients, *n* number of patients with outcome, *OR* odds ratio, *SE* standard error, *TNFi* tumor necrosis factor inhibitor

A similar proportion of overall tofacitinib and overall TNFi initiators with a 12-month visit achieved CDAI-LDA (CDAI ≤10) at month 12 (OR 1.29 [95% CI 0.94, 1.76]; Fig. 1a, b). For the subgroup of non-switchers who remained on therapy to the 12-month visit, there was a higher probability of achieving CDAI-LDA at month 12 in TNFi non-switchers versus tofacitinib non-switchers (OR 1.64 [95% CI 1.17, 2.29]; Fig. 1a, b).

In patients with a 12-month visit, improvements in secondary effectiveness outcomes at month 12 were generally similar for overall tofacitinib and overall TNFi initiators, and for tofacitinib and TNFi non-switchers (Table 3). However, at month 12, mean change from baseline in CDAI was lower in overall tofacitinib versus overall TNFi initiators (mean difference −1.5 [95% CI −2.7, −0.3]; Table 3). The proportion of patients achieving HAQ minimum clinically important difference (decrease of ≥ 0.22 from baseline) at month 12 was lower in overall TNFi versus overall tofacitinib initiators (OR 0.71 [95% CI 0.54, 0.94]; Table 3).

**Table 3:** Secondary effectiveness outcomes at month 12 for overall tofacitinib versus overall TNFi initiators (all initiators and non-switchers^a^) for those with a 12-month visit^b^ (propensity score matched population)

	Overall tofacitinib initiators (*N* = 611)	Overall TNFi initiators (*N* = 611)	Comparison (TNFi vs tofacitinib)
All initiators			
*n*/*N*, predicted probabilities, % (SE)			*Adjusted OR (95% CI)*
CDAI remission (≤ 2.8)^c^	53/572, 9.5 (1.2)	61/565, 10.5 (1.3)	1.11 (0.75, 1.65)
mACR20^d^	114/608, 18.1 (1.5)	109/602, 18.7 (1.6)	1.04 (0.77, 1.41)
mACR50^d^	52/608, 8.4 (1.1)	63/602, 10.7 (1.3)	1.32 (0.89, 1.95)
mACR70^d^	22/608, 3.7 (0.8)	33/602, 5.4 (0.9)	1.49 (0.85, 2.60)
HAQ MCID^e^	174/507, 34.1 (2.1)	135/504, 27.0 (2.0)	0.71 (0.54, 0.94)
Mild pain (≤ 20 mm VAS)^f^	79/472, 17.1 (1.8)	79/476, 16.3 (1.7)	0.94 (0.66, 1.34)
DAS28-4(ESR) LDA/remission (≤ 3.2)^g^	44/265, 16.2 (2.3)	40/232, 17.8 (2.6)	1.12 (0.68, 1.84)
Adjusted mean (SE)			*Adjusted mean difference (95% CI)*
∆CDAI	−3.1 (0.4)	−4.6 (0.4)	−1.5 (−2.7, −0.3)
HAQ	1.0 (0.0)	1.0 (0.0)	0.0 (−0.0, 0.1)
Patient pain (0–100 mm VAS)	43.2 (1.0)	43.5 (1.0)	0.3 (−2.6, 3.1)
Patient fatigue (0–100 mm VAS)	46.9 (1.0)	46.8 (1.0)	−0.0 (−2.8, 2.8)
Morning stiffness (hours)	1.4 (0.1)	1.5 (0.1)	0.1 (−0.2, 0.5)
Non-switchers			
*n*/*N*, predicted probabilities, % (SE)			*Adjusted OR (95% CI)*
CDAI remission (≤ 2.8)^c^	53/445, 12.1 (1.6)	61/398, 15.0 (1.8)	1.29 (0.86, 1.93)
mACR20^d^	114/472, 23.4 (1.9)	109/428, 26.3 (2.1)	1.18 (0.86, 1.62)
mACR50^d^	52/472, 10.8 (1.4)	63/428, 15.1 (1.7)	1.47 (0.99, 2.20)
mACR70^d^	22/472, 4.7 (1.0)	33/428, 7.6 (1.3)	1.66 (0.94, 2.91)
HAQ MCID^e^	174/391, 44.1 (2.5)	135/356, 38.4 (2.6)	0.79 (0.59, 1.06)
Mild pain (VAS; ≤ 20 mm)^f^	79/366, 21.9 (2.2)	79/331, 23.5 (2.3)	1.10 (0.77, 1.58)
DAS28-4(ESR) LDA/remission (≤ 3.2)^g^	44/207, 20.7 (2.8)	40/169, 24.4 (3.4)	1.25 (0.75, 2.07)
Adjusted mean (SE)			*Adjusted mean difference (95% CI)*
∆CDAI	−4.3 (0.5)	−5.6 (0.5)	−1.2 (−2.5, 0.1)
HAQ	0.9 (0.0)	1.0 (0.0)	0.0 (−0.0, 0.1)
Patient pain (0–100 mm VAS)	41.2 (1.1)	41.1 (1.2)	−0.1 (−3.3, 3.2)
Patient fatigue (0–100 mm VAS)	44.8 (1.1)	45.4 (1.2)	0.6 (−2.6, 3.8)
Morning stiffness (hours)	1.3 (0.1)	1.3 (0.1)	0.0 (−0.4, 0.4)

*n*/*N* denotes unadjusted response rates

Predicted probabilities and ORs (binary outcomes), and mean score and mean differences (continuous outcomes) are based on adjusted outcomes following propensity matched scoring. Covariates that remained imbalanced after propensity score matching and were included in the final model for the comparison of tofacitinib versus TNFi were baseline value and age, swollen joint count, and methotrexate use

^a^Non-switchers were defined as patients who initiated tofacitinib or TNFi and continued these treatments to the 12-month visit

^b^12-month visit occurred 10–14 months after the index date

^c^Calculated among those patients with CDAI > 2.8 at baseline

^d^20%, 50%, or 70% improvement in the mACR response criteria

^e^MCID was a decrease of ≥ 0.22 from baseline; calculated among those patients with HAQ > 0.22 at baseline

^f^Calculated among those patients with pain > 20 mm VAS at baseline

^g^Calculated among those patients with DAS28-4(ESR) > 3.2 at baseline

*∆* change from baseline, *CDAI* clinical disease activity index; *CI* confidence interval; *DAS28-4(ESR)* disease Activity Score in 28 joints, erythrocyte sedimentation rate; *HAQ* health assessment questionnaire; *LDA* low disease activity; *mACR* modified American College of Rheumatology; *MCID* minimum clinically important difference; *N* total number of patients; *n* number of patients with outcome; *OR* odds ratio; *SE* standard error; *TNFi* tumor necrosis factor inhibitor; *VAS* visual analog scale

Improvements in primary and secondary effectiveness outcomes for patients with a 6-month visit were generally similar to those with a 12-month visit (Online Resource, Table S3), although there was a higher probability of achieving DAS28-4(ESR) LDA/remission at month 6 in TNFi versus tofacitinib non-switchers (OR 1.57 [95% CI 1.03, 2.38]; Online Resource, Table S3).

#### Tofacitinib monotherapy versus tofacitinib combination therapy

In patients with a 12-month visit, rates of achieving CDAI-LDA at month 12 were similar for tofacitinib monotherapy and tofacitinib combination therapy initiators (OR 1.15 [95% CI 0.70, 1.89]; Online Resource, Table S4). There were no differences in secondary effectiveness outcomes at month 12 between tofacitinib monotherapy and tofacitinib combination therapy initiators (Online Resource, Table S4). Similar results were observed between tofacitinib monotherapy and tofacitinib combination therapy initiators at month 6 (Online Resource, Table S5).

#### TNFi monotherapy versus TNFi combination therapy

No differences in rates of achieving CDAI-LDA were seen between TNFi monotherapy and TNFi combination therapy initiators at month 12 (OR 0.96 [95% CI 0.69, 1.32]; Online Resource, Table S4). Secondary effectiveness outcomes were generally similar between patients initiating TNFi monotherapy and TNFi combination therapy at month 12, with similar trends observed for primary and secondary effectiveness outcomes at month 6 (Online Resource, Tables S4 and S5).

#### Tofacitinib monotherapy versus TNFi combination therapy

At month 12, rates of achieving CDAI-LDA were similar between tofacitinib monotherapy and TNFi combination therapy initiators (OR 1.33 [95% CI 0.81, 2.16]; Online Resource, Table S4). Secondary effectiveness outcomes were generally similar; however, mean patient pain was greater in patients initiating tofacitinib monotherapy versus TNFi combination therapy (mean difference −4.8 [95% CI −9.2, −0.5]). No differences were observed across primary and secondary effectiveness outcomes at month 6 between patients who initiated tofacitinib monotherapy versus TNFi combination therapy (Online Resource, Table S5).

#### Tofacitinib combination therapy versus TNFi combination therapy

Rates of achieving CDAI-LDA at month 12 were similar between tofacitinib combination therapy and TNFi combination therapy initiators (OR 1.23 [95% CI 0.81, 1.87]; Online Resource, Table S4); no differences were observed across the secondary effectiveness outcomes. Similarly, no differences in primary and secondary effectiveness outcomes were observed between tofacitinib combination therapy and TNFi combination therapy initiators at month 6 (Online Resource, Table S5).

### Tofacitinib effectiveness across different lines of therapy

**Fig. 2:**
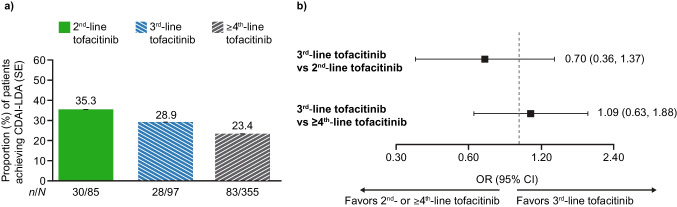
Rates of achieving CDAI-LDA (CDAI ≤ 10) by tofacitinib line of therapy at month 12 in patients with a month 12 visit^a^: **a** response rates and **b** ORs (95% CIs) (unmatched population). Response rates are based on unadjusted outcomes in patients with CDAI > 10 at baseline; N may be less than reported. ORs (95% CIs) are based on models adjusted for tofacitinib exposure, age, duration of RA, race, sex, and baseline CDAI. ^a^12-month visit occurred 10–14 months after the index date. CDAI Clinical Disease Activity Index, CI confidence interval, LDA low disease activity, N total number of patients, n number of patients with outcome, OR odds ratio, RA rheumatoid arthritis, SE standard error

Of 805 patients who initiated tofacitinib and had a 12-month visit, 135 (16.8%), 157 (19.5%), and 513 (63.7%) patients initiated tofacitinib as 2nd-, 3rd-, and ≥ 4th-line therapy, respectively. CDAI-LDA was achieved by 35.3%, 28.9%, and 23.4% of patients initiating tofacitinib as 2nd-, 3rd-, and ≥ 4th-line therapy, respectively (Fig. 2a). ORs (95% CI) were 0.70 (0.36, 1.37) for patients receiving tofacitinib as 3rd-line versus 2nd-line therapy, and 1.09 (0.63, 1.88) for 3rd-line versus ≥ 4th-line therapy (Fig. 2b).

Some differences in secondary effectiveness outcomes were seen at month 12 for patients initiating tofacitinib across different lines of therapy (Online Resource, Tables S6 and S7). Rates of achieving CDAI remission were higher in patients receiving 2nd-line versus 3rd-line tofacitinib (OR 0.37 [95% CI 0.16, 0.83] for 3rd-line vs 2nd-line tofacitinib), but no difference was observed between patients receiving 3rd-line versus ≥ 4th-line tofacitinib. Mean change from baseline in CDAI was greater among patients who initiated tofacitinib as 3rd-line versus ≥ 4th-line therapy (mean difference [95% CI] −2.6 [−4.6, −0.5] for 3rd-line vs ≥ 4th-line tofacitinib), whereas no difference was observed between patients receiving 3rd-line versus 2nd-line tofacitinib. In addition, mean HAQ, patient pain, and patient fatigue were lower in patients who initiated tofacitinib as 2nd-line versus 3rd-line therapy (mean differences 0.2 [95% CI 0.0, 0.3], 8.8 [95% CI 2.4, 15.1], and 11.5 [95% CI 4.7, 18.3], respectively). Mean HAQ and patient pain were also lower in those initiating tofacitinib as 3rd-line versus ≥ 4th-line therapy (mean differences −0.2 [95% CI −0.3, −0.0] and −7.0 [95% CI −12.0, −2.0], respectively).

In general, similar trends were seen in the primary and secondary effectiveness outcomes at month 6 (Online Resource, Tables S8 and S9).

## Discussion

This observational study of data from the US CorEvitas RA Registry evaluated effectiveness of tofacitinib versus TNFi and across tofacitinib as different lines of therapy in patients with RA. While previous real-world studies have compared tofacitinib effectiveness to that of bDMARDs [23, 25, 26, 30–32] and have evaluated tofacitinib prescribing patterns over time [33], we have expanded on these studies by providing an updated analysis (observation period: 2012–2021) of tofacitinib treatment patterns and effectiveness in a large cohort of patients with RA at 12 (3,418 patients) and/or 6 (4,326 patients) months’ follow-up. Notably, we have evaluated real-world effectiveness of tofacitinib initiated in the 2nd line of therapy, which was difficult in the past due to low numbers of patients commencing tofacitinib this early [23]. This analysis showed that improvements in effectiveness outcomes at months 12 and 6 in patients with RA were generally similar in those who initiated tofacitinib monotherapy versus combination therapy; similar improvements in effectiveness outcomes at months 12 and 6 were also observed between those who initiated tofacitinib (monotherapy or combination therapy) versus TNFi combination therapy. Improvements in effectiveness outcomes at months 12 and 6 in patients with RA were also generally similar in those initiating tofacitinib across different lines of therapy; although mean change from baseline in CDAI was lower, and mean HAQ and patient pain were greater at month 12 in those who initiated tofacitinib as ≥ 4th-line versus 3rd-line therapy. In addition, mean HAQ, patient pain and patient fatigue were greater at month 12 in 3rd-line versus 2nd-line tofacitinib initiators, although the sample sizes of these groups were small.

In accordance with previous real-world studies, at baseline, we found that patients initiating tofacitinib versus TNFi had longer disease duration [23, 25, 26, 31, 34] and were less likely to be taking concomitant methotrexate [23, 26, 31]. This may have indicated that tofacitinib initiators may have been more resistant to treatment than the TNFi initiators. We found that 16.8% of tofacitinib initiators commenced tofacitinib as 2nd line of therapy; whereas analysis of data from the US CorEvitas RA Registry at an earlier period (2001–2016), reported that < 10% of tofacitinib initiators commenced tofacitinib as 2nd line of therapy [23] This finding is in keeping with that of Pope et al., who reported that tofacitinib use in earlier lines of therapy (i.e., before receiving bDMARDs or post-1 bDMARD) increased from 2014 to 2017 in patients with RA in Canadian clinical practice [33].

In the present study, we showed that tofacitinib effectiveness was similar with monotherapy versus combination therapy; this observation resembles that of a pooled analysis of tofacitinib RA Phase 3 randomized controlled trials, which revealed no significant differences in efficacy (e.g., ACR20/50/70 and HAQ improvement ≥ 0.22) at month 3 between monotherapy and combination therapy in patients with an inadequate response to DMARDs [35]*.* Similarly, a post hoc analysis of long-term extension studies reported sustained tofacitinib efficacy over 6 years when administered as monotherapy or combination therapy [36]. These findings align with those from a non-interventional study of patients with RA in Australia [34], and with those from a database study of patients with RA in Turkey [37]. We found that effectiveness was generally similar in patients with RA receiving tofacitinib and TNFi, which agrees with findings from real-world RA registries and databases [23, 25]. Notably, we observed that there were generally no differences in effectiveness when comparing tofacitinib monotherapy or combination therapy to TNFi combination therapy. Taken together, our findings suggest that effectiveness may be achieved in patients with RA receiving tofacitinib without the need for concomitant therapies such as methotrexate. However, in a Phase 3b/4 randomized controlled trial which assessed the comparative efficacy of tofacitinib monotherapy, tofacitinib in combination with methotrexate, and adalimumab in combination with methotrexate for the treatment of RA in patients with a previous inadequate response to methotrexate, tofacitinib monotherapy was not shown to be non-inferior to either combination. Tofacitinib in combination with methotrexate was non-inferior to adalimumab in combination with methotrexate [12]. Methotrexate was the most commonly used concomitant csDMARD (from methotrexate, leflunomide, sulfasalazine, hydroxychloroquine, and cyclosporine) with tofacitinib and TNFi initiators (41.4% and 64.3% of overall tofacitinib and TNFi initiators, respectively, had concomitant methotrexate use), although the impact of separate csDMARDs on tofacitinib/TNFi effectiveness was not specifically assessed in this study. However, in a comparative study, leflunomide has been shown to be an effective and safe alternative to methotrexate, when used in combination with tocilizumab [38]. Additionally, a post hoc study of three interventional trials and a real-world study has shown that abatacept in combination with non-methotrexate csDMARDs is an effective and well-tolerated treatment regimen in patients with moderate-to-severe RA, providing similar benefits to those seen with abatacept in combination with methotrexate [39]. Decisions regarding the most appropriate treatment regimen for patients should be made according to individual patient needs.

In this study, we found no difference in the rates of CDAI-LDA at months 12 and 6 between patients initiating tofacitinib across different lines of therapy. Similar results were found in a retrospective cohort study at a center for RA in Colombia, with tofacitinib reported to be effective as both 2nd-line (after csDMARDs) and 3rd-line (after bDMARDs) treatment [40]. We observed lower mean change from baseline in CDAI, and higher mean HAQ, patient pain, and patient fatigue scores at months 12 and 6 in those initiating tofacitinib at later lines of therapy. It is possible that this could reflect more refractory disease, and/or permanent joint damage potentially experienced by patients initiating tofacitinib at later lines of therapy.

This study has several strengths, including the use of longitudinal data collected over a 9-year period (2012–2021) across the US, as well as the fact that the study analyzed several patient-reported and physician-reported measures of wellbeing and disease activity, which may not be accessed through other data sources such as health insurance claims data.

The findings of the study should also be considered in context of its limitations. The CorEvitas RA Registry includes a sample of patients with RA; thus, these results may not be representative of all adults with RA in the US or the rest of the world. The generalizability of the study may be further limited in that only eligible patients who had a visit at month 12 or month 6 were evaluated, where a higher proportion of tofacitinib initiators than TNFi initiators had qualifying visits. Differences between patients with and without qualifying visits were not explored. In the effectiveness analyses of tofacitinib by line of therapy, the sample sizes of the 2nd-line and 3rd-line initiator groups were particularly low. Another limitation is that history of medication use prior to enrolment was derived from what is reported by patients and their current healthcare provider within the registry, and thus is susceptible to recall error. Also, there were no measures of patients’ adherence to treatment that may have impacted the study findings.

In this analysis of data from the US CorEvitas RA Registry, tofacitinib monotherapy demonstrated similar effectiveness when compared with tofacitinib combination therapy or TNFi therapy up to month 12, while differences in some effectiveness outcomes at month 12 were noted with tofacitinib across different lines of therapy. The findings of this study enhance our understanding of the comparative effectiveness of tofacitinib versus TNFi, and of the effectiveness of tofacitinib as different lines of therapy, which may inform future treatment decision-making in patients with RA.

## Supplementary Information

Below is the link to the electronic supplementary material.

Supplementary file1 (PDF 589 KB)

## Data Availability

Data are available from CorEvitas, LLC through a commercial subscription agreement and are not publicly available. No additional data are available from the authors.

## References

[1] Smolen JS, Aletaha D, McInnes IB (2016) Rheumatoid arthritis. Lancet 388:2023–2038. 10.1016/s0140-6736(16)30173-8 27156434 10.1016/S0140-6736(16)30173-8

[2] Hunter TM, Boytsov NN, Zhang X, Schroeder K, Michaud K, Araujo AB (2017) Prevalence of rheumatoid arthritis in the United States adult population in healthcare claims databases, 2004–2014. Rheumatol Int 37:1551–1557. 10.1007/s00296-017-3726-1 28455559 10.1007/s00296-017-3726-1

[3] Finckh A, Gilbert B, Hodkinson B, Bae SC, Thomas R, Deane KD, Alpizar-Rodriguez D, Lauper K (2022) Global epidemiology of rheumatoid arthritis. Nat Rev Rheumatol 18:591–602. 10.1038/s41584-022-00827-y 36068354 10.1038/s41584-022-00827-y

[4] Aletaha D, Smolen JS (2018) Diagnosis and management of rheumatoid arthritis: a review. JAMA 320:1360–1372. 10.1001/jama.2018.13103 30285183 10.1001/jama.2018.13103

[5] Smolen JS, Landewé RBM, Bergstra SA, Kerschbaumer A, Sepriano A, Aletaha D, Caporali R, Edwards CJ, Hyrich KL, Pope JE, de Souza S, Stamm TA, Takeuchi T, Verschueren P, Winthrop KL, Balsa A, Bathon JM, Buch MH, Burmester GR, Buttgereit F, Cardiel MH, Chatzidionysiou K, Codreanu C, Cutolo M, den Broeder AA, El Aoufy K, Finckh A, Fonseca JE, Gottenberg J-E, Haavardsholm EA, Iagnocco A, Lauper K, Li Z, McInnes IB, Mysler EF, Nash P, Poor G, Ristic GG, Rivellese F, Rubbert-Roth A, Schulze-Koops H, Stoilov N, Strangfeld A, van der Helm-van MA, van Duuren E, Vliet Vlieland TPM, Westhovens R, van der Heijde D (2023) EULAR recommendations for the management of rheumatoid arthritis with synthetic and biological disease-modifying antirheumatic drugs: 2022 update. Ann Rheum Dis 82:3–18. 10.1136/ard-2022-223356 36357155 10.1136/ard-2022-223356

[6] Fraenkel L, Bathon JM, England BR, St Clair EW, Arayssi T, Carandang K, Deane KD, Genovese M, Huston KK, Kerr G, Kremer J, Nakamura MC, Russell LA, Singh JA, Smith BJ, Sparks JA, Venkatachalam S, Weinblatt ME, Al-Gibbawi M, Baker JF, Barbour KE, Barton JL, Cappelli L, Chamseddine F, George M, Johnson SR, Kahale L, Karam BS, Khamis AM, Navarro-Millán I, Mirza R, Schwab P, Singh N, Turgunbaev M, Turner AS, Yaacoub S, Akl EA (2021) 2021 American College of Rheumatology guideline for the treatment of rheumatoid arthritis. Arthritis Rheumatol 73:1108–1123. 10.1002/art.41752 34101376 10.1002/art.41752

[7] Yun H, Curtis JR (2013) New methods for determining comparative effectiveness in rheumatoid arthritis. Curr Opin Rheumatol 25:325–333. 10.1097/BOR.0b013e32835fd8c0 23508131 10.1097/BOR.0b013e32835fd8c0 PMC3815611

[8] van Vollenhoven RF, Fleischmann R, Cohen S, Lee EB, García Meijide JA, Wagner S, Forejtova S, Zwillich SH, Gruben D, Koncz T, Wallenstein GV, Krishnaswami S, Bradley JD, Wilkinson B (2012) Tofacitinib or adalimumab versus placebo in rheumatoid arthritis. N Engl J Med 367:508–519. 10.1056/NEJMoa1112072 22873531 10.1056/NEJMoa1112072

[9] Burmester GR, Blanco R, Charles-Schoeman C, Wollenhaupt J, Zerbini C, Benda B, Gruben D, Wallenstein G, Krishnaswami S, Zwillich SH, Koncz T, Soma K, Bradley J, Mebus C, ORAL Step investigators (2013) Tofacitinib (CP-690,550) in combination with methotrexate in patients with active rheumatoid arthritis with an inadequate response to tumour necrosis factor inhibitors: a randomised phase 3 trial. Lancet 381:451–460. 10.1016/S0140-6736(12)61424-X 23294500 10.1016/S0140-6736(12)61424-X

[10] Kremer J, Li Z-G, Hall S, Fleischmann R, Genovese M, Martin-Mola E, Isaacs JD, Gruben D, Wallenstein G, Krishnaswami S, Zwillich SH, Koncz T, Riese R, Bradley J (2013) Tofacitinib in combination with nonbiologic disease-modifying antirheumatic drugs in patients with active rheumatoid arthritis: a randomized trial. Ann Intern Med 159:253–261. 10.7326/0003-4819-159-4-201308200-00006 24026258 10.7326/0003-4819-159-4-201308200-00006

[11] van der Heijde D, Strand V, Tanaka Y, Keystone E, Kremer J, Zerbini CAF, Cardiel MH, Cohen S, Nash P, Song YW, Tegzová D, Gruben D, Wallenstein G, Connell CA, Fleischmann R (2019) Tofacitinib in combination with methotrexate in patients with rheumatoid arthritis: clinical efficacy, radiographic, and safety outcomes from a twenty-four-month, phase III study. Arthritis Rheumatol 71:878–891. 10.1002/art.40803 30666826 10.1002/art.40803 PMC6593705

[12] Fleischmann R, Mysler E, Hall S, Kivitz AJ, Moots RJ, Luo Z, DeMasi R, Soma K, Zhang R, Takiya L, Tatulych S, Mojcik C, Krishnaswami S, Menon S, Smolen JS, on behalf of the ORAL Strategy investigators (2017) Efficacy and safety of tofacitinib monotherapy, tofacitinib with methotrexate, and adalimumab with methotrexate in patients with rheumatoid arthritis (ORAL Strategy): a phase 3b/4, double-blind, head-to-head, randomised controlled trial. Lancet 390:457–468. 10.1016/S0140-6736(17)31618-5 28629665 10.1016/S0140-6736(17)31618-5

[13] Lee EB, Fleischmann R, Hall S, Wilkinson B, Bradley J, Gruben D, Koncz T, Krishnaswami S, Wallenstein GV, Zang C, Zwillich SH, van Vollenhoven RF (2014) Tofacitinib versus methotrexate in rheumatoid arthritis. N Engl J Med 370:2377–2386. 10.1056/NEJMoa1310476 24941177 10.1056/NEJMoa1310476

[14] Cohen SB, Pope J, Haraoui B, Irazoque-Palazuelos F, Korkosz M, Diehl A, Rivas JL, Lukic T, Liu S, Stockert L, Iikuni N, Keystone EC (2019) Methotrexate withdrawal in patients with rheumatoid arthritis who achieve low disease activity with tofacitinib modified-release 11 mg once daily plus methotrexate (ORAL Shift): a randomised, phase 3b/4, non-inferiority trial. Lancet Rheumatol 1:E23–E34. 10.1016/S2665-9913(19)30005-0 38229356 10.1016/S2665-9913(19)30005-0

[15] Fleischmann R, Kremer J, Cush J, Schulze-Koops H, Connell CA, Bradley JD, Gruben D, Wallenstein GV, Zwillich SH, Kanik KS, Solo Investigators ORAL (2012) Placebo-controlled trial of tofacitinib monotherapy in rheumatoid arthritis. N Engl J Med 367:495–507. 10.1056/NEJMoa1109071 22873530 10.1056/NEJMoa1109071

[16] Wollenhaupt J, Silverfield J, Lee EB, Wood SP, Terry K, Nakamura H, Kwok K, Anisfeld A, Nduaka C, Riese R, Wang L (2014) Tofacitinib, an oral Janus kinase inhibitor, in the treatment of rheumatoid arthritis: safety and efficacy in open-label, long-term extension studies up to 6 years. Arthritis Rheumatol 66:S375 10.3899/jrheum.130683 24692527

[17] Yamanaka H, Tanaka Y, Takeuchi T, Sugiyama N, Yuasa H, Toyoizumi S, Morishima Y, Hirose T, Zwillich S (2016) Tofacitinib, an oral Janus kinase inhibitor, as monotherapy or with background methotrexate, in Japanese patients with rheumatoid arthritis: an open-label, long-term extension study. Arthritis Res Ther 18:34. 10.1186/s13075-016-0932-2 26818974 10.1186/s13075-016-0932-2 PMC4730592

[18] Wollenhaupt J, Lee EB, Curtis JR, Silverfield J, Terry K, Soma K, Mojcik C, DeMasi R, Strengholt S, Kwok K, Lazariciu I, Wang L, Cohen S (2019) Safety and efficacy of tofacitinib for up to 9.5 years in the treatment of rheumatoid arthritis: final results of a global, open-label, long-term extension study. Arthritis Res Ther 21:89. 10.1186/s13075-019-1866-2 30953540 10.1186/s13075-019-1866-2 PMC6451219

[19] Ytterberg SR, Bhatt DL, Mikuls TR, Koch GG, Fleischmann R, Rivas JL, Germino R, Menon S, Sun Y, Wang C, Shapiro AB, Kanik KS, Connell CA, Surveillance Investigators ORAL (2022) Cardiovascular and cancer risk with tofacitinib in rheumatoid arthritis. N Engl J Med 386:316–326. 10.1056/NEJMoa2109927 35081280 10.1056/NEJMoa2109927

[20] US Food and Drug Administration (2024) XELJANZ^®^ (tofacitinib): highlights of prescribing information. https://labeling.pfizer.com/showlabeling.aspx?id=959. Accessed: February 26, 2024

[21] Blonde L, Khunti K, Harris SB, Meizinger C, Skolnik NS (2018) Interpretation and impact of real-world clinical data for the practicing clinician. Adv Ther 35:1763–1774. 10.1007/s12325-018-0805-y 30357570 10.1007/s12325-018-0805-y PMC6223979

[22] Harnett J, Gerber R, Gruben D, Koenig AS, Chen C (2016) Evaluation of real-world experience with tofacitinib compared with adalimumab, etanercept, and abatacept in RA patients with 1 previous biologic DMARD: data from a U.S. administrative claims database. J Manag Care Spec Pharm 22:1457–1471. 10.18553/jmcp.2016.22.12.1457 27882833 10.18553/jmcp.2016.22.12.1457 PMC10397820

[23] Reed GW, Gerber RA, Shan Y, Takiya L, Dandreo KJ, Gruben D, Kremer J, Wallenstein G (2019) Real-world comparative effectiveness of tofacitinib and tumor necrosis factor inhibitors as monotherapy and combination therapy for treatment of rheumatoid arthritis. Rheumatol Ther 6:573–586. 10.1007/s40744-019-00177-4 31707603 10.1007/s40744-019-00177-4 PMC6858427

[24] Pappas DA, St John G, Etzel CJ, Fiore S, Blachley T, Kimura T, Punekar R, Emeanuru K, Choi J, Boklage S, Kremer JM (2021) Comparative effectiveness of first-line tumour necrosis factor inhibitor versus non-tumour necrosis factor inhibitor biologics and targeted synthetic agents in patients with rheumatoid arthritis: results from a large US registry study. Ann Rheum Dis 80:96–102. 10.1136/annrheumdis-2020-217209 32719038 10.1136/annrheumdis-2020-217209 PMC7788059

[25] Movahedi M, Cesta A, Li X, Keystone EC, Bombardier C (2022) Physician- and patient-reported effectiveness are similar for tofacitinib and TNFi in rheumatoid arthritis: data from a rheumatoid arthritis registry. J Rheumatol 49:447–453. 10.3899/jrheum.211066 35169051 10.3899/jrheum.211066

[26] Barbulescu A, Askling J, Chatzidionysiou K, Forsblad-d’Elia H, Kastbom A, Lindström U, Turesson C, Frisell T (2022) Effectiveness of baricitinib and tofacitinib compared with bDMARDs in RA: results from a cohort study using nationwide Swedish register data. Rheumatology (Oxford) 61:3952–3962. 10.1093/rheumatology/keac068 35134119 10.1093/rheumatology/keac068 PMC9536798

[27] Kremer J (2005) The CORRONA database. Ann Rheum Dis 64(Suppl 4):iv37–iv41. 10.1136/ard.2005.043497 16239384 10.1136/ard.2005.043497 PMC1766903

[28] Kremer JM (2016) The Corrona US registry of rheumatic and autoimmune diseases. Clin Exp Rheumatol 34:S96-99 27762197

[29] Greenberg JD, Harrold LR, Bentley MJ, Kremer J, Reed G, Strand V (2009) Evaluation of composite measures of treatment response without acute-phase reactants in patients with rheumatoid arthritis. Rheumatology (Oxford) 48:686–690. 10.1093/rheumatology/kep054 19395544 10.1093/rheumatology/kep054 PMC2722796

[30] Machado MAÁ, Moura CS, Guerra SF, Curtis JR, Abrahamowicz M, Bernatsky S (2018) Effectiveness and safety of tofacitinib in rheumatoid arthritis: a cohort study. Arthritis Res Ther 20:60. 10.1186/s13075-018-1539-6 29566769 10.1186/s13075-018-1539-6 PMC5865387

[31] Finckh A, Tellenbach C, Herzog L, Scherer A, Moeller B, Ciurea A, von Muehlenen I, Gabay C, Kyburz D, Brulhart L, Müller R, Hasler P, Zufferey P, Physicians and Patients of the SCQM (2020) Comparative effectiveness of antitumour necrosis factor agents, biologics with an alternative mode of action and tofacitinib in an observational cohort of patients with rheumatoid arthritis in Switzerland. RMD Open 6:e001174. 10.1136/rmdopen-2020-001174 32385143 10.1136/rmdopen-2020-001174 PMC7299517

[32] Wei W, Knapp K, Wang L, Chen CI, Craig GL, Ferguson K, Schwartzman S (2017) Treatment persistence and clinical outcomes of tumor necrosis factor inhibitor cycling or switching to a new mechanism of action therapy: real-world observational study of rheumatoid arthritis patients in the United States with prior tumor necrosis factor inhibitor therapy. Adv Ther 34:1936–1952. 10.1007/s12325-017-0578-8 28674959 10.1007/s12325-017-0578-8 PMC5565674

[33] Pope J, Bessette L, Jones N, Fallon L, Woolcott J, Gruben D, Crooks M, Gold D, Haraoui B (2020) Experience with tofacitinib in Canada: patient characteristics and treatment patterns in rheumatoid arthritis over 3 years. Rheumatology (Oxford) 59:568–574. 10.1093/rheumatology/kez324 31410469 10.1093/rheumatology/kez324

[34] Bird P, Littlejohn G, Butcher B, Smith T, O’Sullivan C, Witcombe D, Griffiths H (2022) Real-world evaluation of effectiveness, persistence, and usage patterns of monotherapy and combination therapy tofacitinib in treatment of rheumatoid arthritis in Australia. Clin Rheumatol 41:53–62. 10.1007/s10067-021-05853-x 34370130 10.1007/s10067-021-05853-x PMC8724080

[35] Keystone E, Fleischmann R, van Vollenhoven R, Kremer J, Gruben D, Bradley J, Riese R, Mebus C, Wallenstein G, Zwillich SH, Benda B, Krishnaswami S (2013) THU0228 Tofacitinib, an oral Janus kinase inhibitor: post-hoc analyses of efficacy and safety of monotherapy versus combination therapy in a Phase 3 rheumatoid arthritis population. Ann Rheum Dis 72:A242

[36] Fleischmann R, Wollenhaupt J, Takiya L, Maniccia A, Kwok K, Wang L, van Vollenhoven RF (2017) Safety and maintenance of response for tofacitinib monotherapy and combination therapy in rheumatoid arthritis: an analysis of pooled data from open-label long-term extension studies. RMD Open 3:e000491. 10.1136/rmdopen-2017-000491 29435359 10.1136/rmdopen-2017-000491 PMC5761286

[37] Bilgin E, Ceylan F, Duran E, Farisogullari B, Bolek EC, Yardimci GK, Kilic L, Akdogan A, Karadag O, Apras Bilgen SS, Kiraz S, Ertenli AI, Kalyoncu U (2021) Efficacy, retention, and safety of tofacitinib in real-life: Hur-bio monocentric experience. Turk J Med Sci 51:297–308. 10.3906/sag-2007-123 32979899 10.3906/sag-2007-123 PMC7991862

[38] Narváez J, Díaz-Torné C, Magallares B, Hernández MV, Reina D, Corominas H, Sanmartí R, de la Serna AR, Llobet JM, Nolla JM (2015) Comparative effectiveness of tocilizumab with either methotrexate or leflunomide in the treatment of rheumatoid arthritis. PLoS One 10:e0123392. 10.1371/journal.pone.0123392 25830224 10.1371/journal.pone.0123392 PMC4382296

[39] Alten R, Burkhardt H, Feist E, Krüger K, Rech J, Rubbert-Roth A, Voll RE, Elbez Y, Rauch C (2018) Abatacept used in combination with non-methotrexate disease-modifying antirheumatic drugs: a descriptive analysis of data from interventional trials and the real-world setting. Arthritis Res Ther 20:1. 10.1186/s13075-017-1488-5 29329602 10.1186/s13075-017-1488-5 PMC5795278

[40] Santos-Moreno P, Martinez S, Ibata L, Villarreal L, Rodríguez-Florido F, Rivero M, Rojas-Villarraga A, Galarza-Maldonado C (2022) Is tofacitinib effectiveness in patients with rheumatoid arthritis better after conventional than after biological therapy? - A cohort study in a Colombian population. Biologics 16:107–117. 10.2147/btt.S361164 35860386 10.2147/BTT.S361164 PMC9289171

