# Supplementary material for: Effectiveness of tofacitinib versus tumor necrosis factor inhibitors and in those receiving tofacitinib as different lines of therapy in patients with rheumatoid arthritis: results from the United States CorEvitas Rheumatoid Arthritis Registry
Source: Clin Rheumatol. 2024 Dec 20;44(2):635–48. doi: 10.1007/s10067-024-07245-3 (PMC11775051; doi:10.1007/s10067-024-07245-3)
Supplement: Supplementary file 1 — Supplementary file1 (PDF 589 KB) [file 10067_2024_7245_MOESM1_ESM.pdf]

Clinical Rheumatology

Effectiveness of tofacitinib versus tumor necrosis factor inhibitors and in those receiving tofacitinib as different lines of therapy in patients with rheumatoid arthritis: results from the United States CorEvitas Rheumatoid Arthritis Registry

Leslie R. Harrold[1, 2], Clifton O. Bingham[3], Janet E. Pope[4], Jacqueline O'Brien[1], Page C. Moore[1], Carla Roberts-Toler[1], Miao Yu[1], Lindsay L. Sweet[5], Ahmed Shelbaya[6], Karim R. Masri[5]

[1]CorEvitas, LLC, Waltham, MA, USA; [2]Department of Medicine, Division of Rheumatology, University of Massachusetts Chan Medical School, Worcester, MA, USA; [3]Division of Rheumatology, Department of Medicine, Johns Hopkins University School of Medicine, Baltimore, MD, USA; [4]Medicine, Division Rheumatology, Western University, London, Ontario, Canada; [5]Pfizer Inc, Collegeville, PA, USA; [6]Pfizer Inc, New York, NY, USA

Correspondence: Dr Ahmed Shelbaya, Pfizer Inc, 66 Hudson Boulevard, New York, NY, 10001, USA. Ahmed.Shelbaya@pfizer.com

Supplemental Material

Fig. S1 Patient selection flowchart for tofacitinib and TNFi initiators with a 6- and 12-month visit[a]

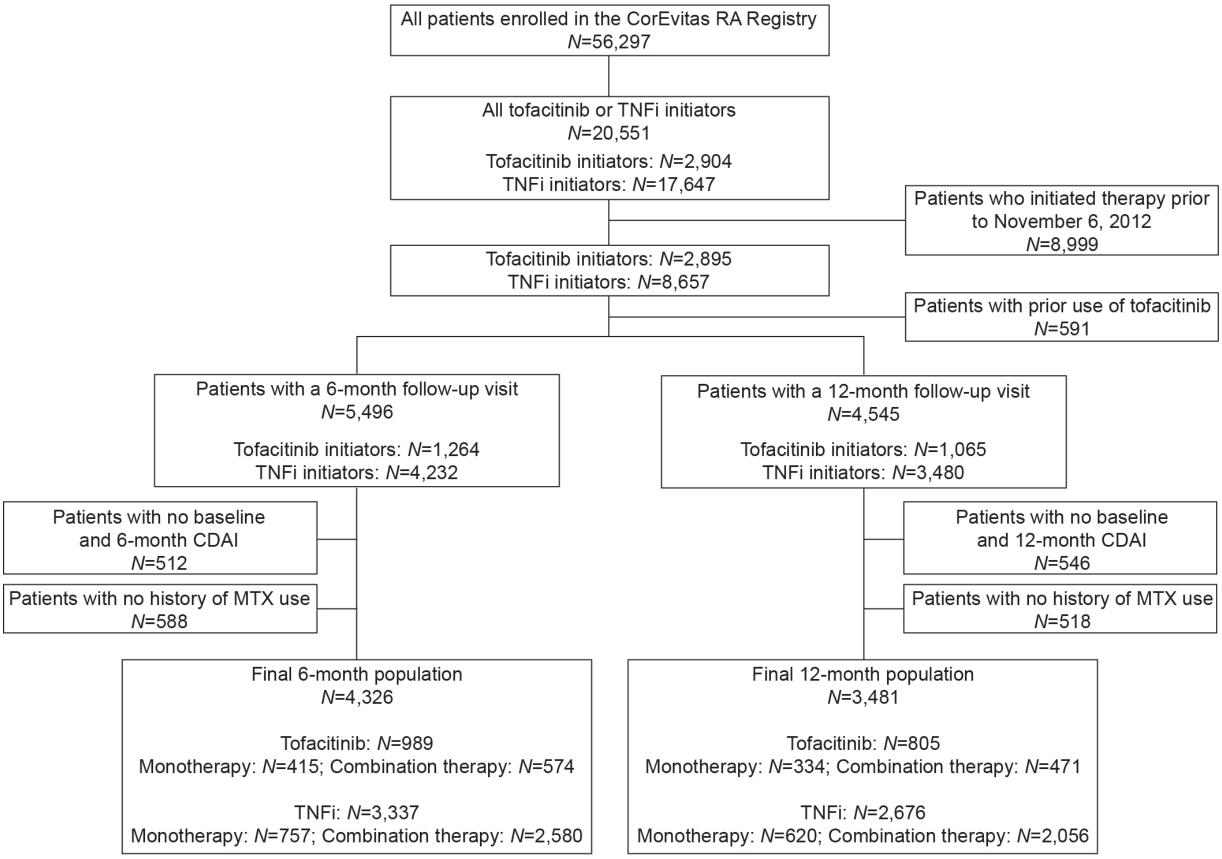

[a]12-month visit occurred 10–14 months after the index date; 6-month visit occurred 4–8 months after the index date. *CDAI* Clinical Disease Activity Index, *MTX* methotrexate, *N* number of patients, *RA* rheumatoid arthritis, *TNFi* tumor necrosis factor inhibitor

Table S1 Demographics and baseline characteristics of overall tofacitinib and overall TNFi initiators with a 6-month visit[a] (unmatched population)

	**Overall tofacitinib initiators[b] (*N*=989)**	**Overall TNFi initiators[b] (*N*=3,337)**	**Standardized difference**
Age, mean (SD)	60.5 (11.8)	58.4 (12.8)	0.172
Female, *n* (%)	804 (81.5)	2,634 (78.9)	0.063
Race, *n* (%)			
White	846 (86.9)	2,716 (82.2)	0.130
Hispanic	67 (6.9)	272 (8.2)	0.051
Black	42 (4.3)	212 (6.4)	0.093
Asian	6 (0.6)	55 (1.7)	0.099
Other	13 (1.3)	50 (1.5)	0.015
Medical insurance, *n* (%)			
None	9 (0.9)	66 (2.0)	0.090
Medicaid	59 (6.0)	184 (5.5)	0.019
Medicare	427 (43.2)	1,184 (35.5)	0.158
Private	668 (67.5)	2,350 (70.4)	0.062
Current smoker, *n* (%)	188 (19.2)	621 (18.9)	0.010
BMI, kg/m^2, mean (SD)	30.1 (7.3)	30.6 (7.5)	0.075
History of bDMARD use, *n* (%)			
bDMARD naïve	172 (17.4)	1,725 (51.7)	0.773
1 prior bDMARD	195 (19.7)	960 (28.8)	0.212
2 prior bDMARDs	196 (19.8)	308 (9.2)	0.304
≥3 prior bDMARDs	426 (43.1)	344 (10.3)	0.797
Prednisone use, *n* (%)	285 (28.8)	917 (27.5)	0.030
Methotrexate use, *n* (%)	414 (41.9)	2,130 (63.8)	0.451
History of comorbidities, *n* (%)			
Cardiovascular disease[c]	174 (17.6)	399 (12.0)	0.159
Hypertension	340 (34.4)	1,096 (32.8)	0.033
Malignancy[d]	88 (8.9)	205 (6.1)	0.105
**RA-related characteristics**			
Duration of RA, years, mean (SD)	13.8 (10.4)	9.2 (9.5)	0.465
CDAI			
Mean (SD)	18.5 (13.1)	19.4 (13.9)	0.068
Median (Q1, Q3)	16.0 (8.0, 26.0)	17.0 (9.0, 27.0)	
CDAI, *n* (%)			
Remission (CDAI ≤2.8)	75 (7.6)	281 (8.4)	0.031
LDA (2.8< CDAI ≤10)	242 (24.5)	688 (20.6)	0.092
MoDA (10< CDAI ≤22)	334 (33.8)	1,170 (35.1)	0.027
HDA (22< CDAI)	338 (34.2)	1,198 (35.9)	0.036
Tender joint count (28), mean (SD)	6.5 (7.0)	6.7 (7.1)	0.032
Swollen joint count (28), mean (SD)	4.4 (4.9)	4.9 (5.3)	0.094

	**Overall tofacitinib initiators[b] (*N*=989)**	**Overall TNFi initiators[b] (*N*=3,337)**	**Standardized difference**
DAS28-4(ESR), mean (SD)	4.2 (1.6)	4.1 (1.6)	0.078
Physician Global Assessment, mean (SD)	30.3 (21.7)	33.2 (23.4)	0.131
Patient Global Assessment, mean (SD)	45.6 (27.1)	44.9 (27.2)	0.026
Patient pain assessment (0–100 mm VAS), mean (SD)	49.1 (28.5)	48.1 (28.8)	0.033
Patient fatigue assessment (0–100 mm VAS), mean (SD)	49.9 (30.1)	48.0 (29.7)	0.062
HAQ (0–3), mean (SD)	1.1 (0.7)	1.0 (0.7)	0.154
mHAQ, mean (SD)	0.6 (0.5)	0.5 (0.5)	0.125
Patients with morning stiffness, *n* (%)	849 (86.2)	2,849 (85.9)	0.008
Morning stiffness duration, *n* (%)[e]			
<30 minutes	144 (17.0)	496 (17.4)	0.010
30–59 minutes	163 (19.2)	557 (19.6)	0.007
60–119 minutes	240 (28.3)	751 (26.4)	0.040
≥120 minutes	296 (34.9)	1,025 (36.0)	0.019

N for each specific outcome may vary

Data shown are not propensity score matched

[a]6-month visit occurred 4–8 months after the index date

[b]Initiators included all patients who initiated tofacitinib or TNFi, including non-switchers

[c]Included carotid artery disease, acute coronary syndrome, cardiac arrest, congestive heart failure, coronary artery disease, myocardial infarction, revascularization procedures (coronary artery bypass grafting, coronary artery stents, or percutaneous coronary intervention), stroke, transient ischemic attack, unstable angina, ventricular arrhythmia, and other cardiovascular diseases

[d]History of lung cancer, breast cancer, lymphoma, skin cancer (basal, melanoma, and squamous), or other cancer

[e]In patients who reported morning stiffness

bDMARD biologic disease-modifying antirheumatic drug, *BMI* body mass index, *CDAI* Clinical Disease Activity Index, *DAS28-4(ESR)* Disease Activity Score in 28 joints, erythrocyte sedimentation rate, *HAQ* Health Assessment Questionnaire, *HDA* high disease activity, *LDA* low disease activity, *MoDA* moderate disease activity, *mHAQ* modified Health Assessment Questionnaire, *N* total number of patients, *n* number of patients with outcome, *Q* quartile, *RA* rheumatoid arthritis, *SD* standard deviation, *TNFi* tumor necrosis factor inhibitor, *VAS* Visual Analog Scale

Table S2 Demographics and baseline characteristics of overall tofacitinib and overall TNFi initiators with a 6-month visit[a] (propensity score matched population)

	**Overall tofacitinib initiators[b] (*N*=735)**	**Overall TNFi initiators[b] (*N*=735)**	**Standardized difference**
Age, mean (SD)	60.6 (11.7)	61.9 (11.4)	0.110
Female, *n* (%)	609 (82.9)	613 (83.4)	0.015
Race, *n* (%)			
White	650 (88.4)	678 (92.2)	0.129
Hispanic	46 (6.3)	33 (4.5)	0.078
Black	24 (3.3)	16 (2.2)	0.067
Asian	-[c]	-[d]	-
Other	-[c]	-[c]	0.026
Medical insurance, *n* (%)			
None	-[d]	-[c]	-
Medicaid	-[c]	-[c]	0.061
Medicare	320 (43.5)	354 (48.2)	0.093
Private	510 (69.4)	485 (66.0)	0.073
Current smoker, *n* (%)	131 (17.8)	129 (17.6)	0.007
BMI, kg/m^2, mean (SD)	30.1 (7.3)	30.2 (7.4)	0.023
History of bDMARD use, *n* (%)			
bDMARD naïve	149 (20.3)	149 (20.3)	-
1 prior bDMARD	173 (23.5)	173 (23.5)	-
2 prior bDMARDs	144 (19.6)	144 (19.6)	-
≥3 prior bDMARDs	269 (36.6)	269 (36.6)	-
Prednisone use, *n* (%)	210 (28.6)	213 (29.0)	0.009
Methotrexate use, *n* (%)	338 (46.0)	286 (38.9)	0.144
History of comorbidities, *n* (%)			
Cardiovascular disease[e]	124 (16.9)	108 (14.7)	0.060
Hypertension	247 (33.6)	269 (36.6)	0.063
Malignancy[f]	66 (9.0)	53 (7.2)	0.065
**RA-related characteristics**			
Duration of RA, years, mean (SD)	13.1 (10.0)	14.6 (10.8)	0.145
CDAI			
Mean (SD)	18.9 (13.4)	19.2 (13.5)	0.024
Median (Q1, Q3)	16.0 (8.5, 26.0)	17.0 (8.2, 27.0)	
CDAI, *n* (%)			
Remission (CDAI ≤2.8)	49 (6.7)	55 (7.5)	0.032
LDA (2.8< CDAI ≤10)	184 (25.0)	167 (22.7)	0.054
MoDA (10< CDAI ≤22)	246 (33.5)	239 (32.5)	0.020
HDA (22< CDAI)	256 (34.8)	274 (37.3)	0.051
Tender joint count (28), mean (SD)	6.7 (7.3)	6.8 (7.3)	0.006
Swollen joint count (28), mean (SD)	4.6 (5.2)	4.5 (4.8)	0.025

	Overall tofacitinib initiators[b] (*N*=735)	Overall TNFi initiators[b] (*N*=735)	Standardized difference
DAS28-4(ESR), mean (SD)	4.2 (1.6)	4.0 (1.6)	0.097
Physician Global Assessment, mean (SD)	30.5 (21.9)	32.3 (23.0)	0.078
Patient Global Assessment, mean (SD)	44.9 (26.5)	47.2 (26.2)	0.087
Patient pain assessment (0–100 mm VAS), mean (SD)	48.5 (28.3)	50.5 (28.1)	0.071
Patient fatigue assessment (0–100 mm VAS), mean (SD)	49.9 (30.2)	50.8 (29.2)	0.031
HAQ (0–3), mean (SD)	1.1 (0.7)	1.1 (0.7)	0.078
mHAQ, mean (SD)	0.6 (0.5)	0.6 (0.5)	0.023
Patients with morning stiffness, *n* (%)	634 (86.3)	652 (88.7)	0.074
Morning stiffness duration, *n* (%)[g]			
<30 minutes	112 (17.7)	104 (16.0)	0.030
30–59 minutes	126 (19.9)	128 (19.6)	0.008
60–119 minutes	181 (28.5)	178 (27.3)	0.008
≥120 minutes	212 (33.4)	239 (36.7)	0.082

N for each specific outcome may vary

Overall tofacitinib and overall TNFi initiators were propensity matched by sex, age, duration of RA, CDAI, smoking status, work status, insurance, methotrexate use, HAQ, patient-reported pain, patient-reported fatigue, morning stiffness, serum positivity, history of non-TNFi use, and race

[a]6-month visit occurred 4–8 months after the index date

[b]Initiators included all patients who initiated tofacitinib or TNFi, including non-switchers

[c]Cell value ≥5 patients but suppressed to minimize risk of reidentification

[d]Cell values of <5 patients have been suppressed to minimize risk of reidentification

[e]Included carotid artery disease, acute coronary syndrome, cardiac arrest, congestive heart failure, coronary artery disease, myocardial infarction, revascularization procedures (coronary artery bypass grafting, coronary artery stents, or percutaneous coronary intervention), stroke, transient ischemic attack, unstable angina, ventricular arrhythmia, and other cardiovascular diseases

[f]History of lung cancer, breast cancer, lymphoma, skin cancer (basal, melanoma, and squamous), or other cancer

[g]In patients who reported morning stiffness

bDMARD biologic disease-modifying antirheumatic drug, *BMI* body mass index, *CDAI* Clinical Disease Activity Index, *DAS28-4(ESR)* Disease Activity Score in 28 joints, erythrocyte sedimentation rate, *HAQ* Health Assessment Questionnaire, *HDA* high disease activity, *LDA* low disease activity, *MoDA* moderate

disease activity, *mHAQ* modified Health Assessment Questionnaire, *N* total number of patients, *n* number of patients with outcome, *Q* quartile, *RA* rheumatoid arthritis, *SD* standard deviation, *TNFi* tumor necrosis factor inhibitor, *VAS* Visual Analog Scale

Table S3 Primary and secondary effectiveness outcomes at month 6 for tofacitinib versus TNFi initiators (all initiators and non-switchers[a]) for those with a 6-month visit[b] (propensity score matched population)

	**Tofacitinib initiators (*N*=735)**	**TNFi initiators (*N*=735)**	**Comparison (TNFi vs tofacitinib)**
**All initiators**			
n/*N*, predicted probabilities, % (SE)			*Adjusted OR (95% CI)*
CDAI-LDA (2.8< CDAI ≤10)[c]	129/502, 25.7 (2.0)	140/513, 27.3 (2.0)	1.09 (0.82, 1.44)
CDAI remission (≤2.8)[d]	61/686, 9.0 (1.1)	71/680, 10.3 (1.2)	1.16 (0.80, 1.66)
mACR20[e]	138/731, 18.9 (1.5)	143/730, 19.5 (1.5)	1.04 (0.80, 1.35)
mACR50[e]	73/731, 10.1 (1.1)	87/730, 11.8 (1.2)	1.20 (0.86, 1.67)
mACR70[e]	33/731, 4.6 (0.8)	32/730, 4.3 (0.7)	0.93 (0.57, 1.54)
HAQ MCID[f]	197/614, 31.8 (1.9)	213/618, 34.7 (1.9)	1.14 (0.90, 1.45)
Mild pain (VAS; ≤20 mm)[g]	98/564, 17.5 (1.6)	102/586, 17.3 (1.6)	0.99 (0.73, 1.34)
DAS28-4(ESR) LDA/remission (≤3.2)[h]	54/314, 17.3 (2.1)	64/279, 22.8 (2.5)	1.42 (0.94, 2.15)
Adjusted mean (SE)			*Adjusted mean difference (95% CI)*
ΔCDAI	-4.0 (0.4)	-3.8 (0.4)	0.2 (-0.9, 1.3)
HAQ	1.1 (0.0)	1.0 (0.0)	-0.0 (-0.1, 0.0)
Patient pain (0–100 mm VAS)	44.0 (0.9)	43.9 (0.9)	-0.1 (-2.7, 2.5)
Patient fatigue (0–100 mm VAS)	46.5 (0.9)	48.2 (0.9)	1.7 (-0.7, 4.1)
Morning stiffness (minutes)	1.4 (0.10)	1.5 (0.10)	0.1 (-0.2, 0.4)
**Non-switchers**			
n/*N*, predicted probabilities, % (SE)			*Adjusted OR (95% CI)*
CDAI-LDA (2.8< CDAI ≤10)[c]	129/442, 29.2 (2.2)	140/443, 31.6 (2.2)	1.12 (0.84, 1.50)
CDAI remission (≤2.8)[d]	61/602, 10.3 (1.2)	71/593, 11.8 (1.3)	1.18 (0.82, 1.70)
mACR20[e]	138/643, 21.6 (1.6)	143/639, 22.2 (1.6)	1.04 (0.80, 1.35)
mACR50[e]	73/643, 11.5 (1.3)	87/639, 13.5 (1.3)	1.21 (0.86, 1.69)

	Tofacitinib initiators (*N*=735)	TNFi initiators (*N*=735)	Comparison (TNFi vs tofacitinib)
mACR70[e]	33/643, 5.2 (0.9)	32/639, 4.9 (0.9)	0.94 (0.57, 1.56)
HAQ MCID[f]	197/543, 36.0 (2.1)	213/535, 40.1 (2.1)	1.19 (0.93, 1.52)
Mild pain (VAS; ≤20 mm)[g]	98/495, 19.9 (1.8)	102/511, 19.9 (1.8)	1.00 (0.73, 1.36)
DAS28-4(ESR) LDA/remission (≤3.2)[h]	54/283, 19.2 (2.3)	64/236, 27.0 (2.9)	1.57 (1.03, 2.38)
Adjusted mean (SE)			*Adjusted mean difference (95% CI)*
ΔCDAI	-4.5 (0.4)	-4.3 (0.4)	0.3 (-0.9, 1.4)
HAQ	1.0 (0.0)	1.0 (0.0)	-0.0 (-0.1, 0.0)
Patient pain (0–100 mm VAS)	43.0 (1.0)	43.1 (1.0)	0.2 (-2.5, 2.9)
Patient fatigue (0–100 mm VAS)	45.9 (0.9)	47.4 (0.9)	1.6 (-1.0, 4.1)
Morning stiffness (hours)	1.4 (0.10)	1.4 (0.10)	0.1 (-0.2, 0.4)

n/N denotes unadjusted response rates

Predicted probabilities and ORs (binary outcomes), and mean score and mean differences (continuous outcomes) are based on adjusted outcomes following propensity score matching. Covariates that remained imbalanced after propensity score matching and were included in the final model for the comparison of tofacitinib versus TNFi were baseline value and age, duration of RA, methotrexate use and race

[a]Non-switchers were defined as patients who initiated tofacitinib or TNFi and continued these treatments to the 12- or 6-month visit

[b]6-month visit occurred 4–8 months after the index date.

[c]Calculated among those patients with CDAI >10 at baseline

[d]Calculated among those patients with CDAI >2.8 at baseline

[e]20%, 50%, or 70% improvement in the mACR response criteria

[f]MCID was a decrease of ≥0.22 from baseline; calculated among those patients with HAQ >0.22 at baseline

[g]Calculated among those patients with pain >20 mm VAS at baseline

[h]Calculated among those patients with DAS28-4(ESR) >3.2 at baseline

Δ change from baseline, *CDAI* Clinical Disease Activity Index, *CI* confidence interval, *DAS28-4(ESR)* Disease Activity Score in 28 joints, erythrocyte sedimentation rate, *HAQ* Health Assessment Questionnaire, *LDA* low disease activity, *mACR* modified American College of Rheumatology, *MCID* minimum clinically important difference, *N* total number of patients, *n* number of patients with outcome, *OR* odds ratio, *SD* standard deviation, *SE* standard error, *TNFi* tumor necrosis factor inhibitor, *VAS* Visual Analog Scale

Table S4 Primary and secondary effectiveness outcomes by treatment regimen at month 12 in those with a 12-month visit[a] (propensity score matched population)

	**Tofacitinib monotherapy (*N*=277)**	**Tofacitinib combination therapy (*N*=277)**	**Comparison[b] (Tofacitinib combination therapy vs tofacitinib monotherapy)**	**TNFi monotherapy (*N*=536)**	**TNFi combination therapy (*N*=536)**	**Comparison[c] (TNFi combination therapy vs TNFi monotherapy)**
**All initiators**						
n/*N*, predicted probabilities, % (SE)			*Adjusted OR (95% CI)*			*Adjusted OR (95% CI)*
CDAI-LDA (CDAI ≤10)[d]	44/180, 23.8 (3.1)	48/176, 26.4 (3.4)	1.15 (0.70, 1.89)	123/362, 35.6 (2.4)	129/356, 34.6 (2.4)	0.96 (0.69, 1.32)
CDAI remission (≤2.8)[e]	23/259, 8.8 (1.8)	24/251, 8.1 (1.7)	0.92 (0.49, 1.75)	55/474, 12.2 (1.5)	56/485, 11.1 (1.4)	0.90 (0.60, 1.35)
mACR20[f]	46/272, 16.8 (2.3)	43/267, 15.2 (2.2)	0.89 (0.56, 1.42)	111/531, 20.4 (1.7)	107/533, 20.5 (1.7)	1.01 (0.74, 1.38)
mACR50[f]	20/272, 7.1 (1.6)	19/267, 6.6 (1.5)	0.91 (0.46, 1.80)	59/531, 10.9 (1.3)	59/533, 11.2 (1.4)	1.03 (0.69, 1.52)
mACR70[f]	12/272, 4.6 (1.3)	8/267, 2.5 (1.0)	0.54 (0.19, 1.48)	34/531, 6.3 (1.0)	26/533, 5.0 (1.0)	0.78 (0.46, 1.33)
HAQ MCID[g]	73/226, 32.4 (3.1)	75/221, 34.1 (3.2)	1.08 (0.72, 1.61)	129/429, 30.5 (2.2)	143/452, 31.2 (2.2)	1.04 (0.77, 1.39)
Mild pain (VAS; ≤20 mm)[h]	30/214, 14.8 (2.5)	27/209, 13.5 (2.4)	0.90 (0.50, 1.60)	73/409, 18.2 (1.9)	71/428, 16.4 (1.8)	0.87 (0.60, 1.26)
DAS28-4(ESR) LDA/remission (≤3.2)[i]	14/112, 11.7 (3.0)	16/108, 15.4 (3.5)	1.38 (0.62, 3.07)	39/204, 20.0 (2.8)	45/205, 20.8 (2.8)	1.05 (0.64, 1.74)
Adjusted mean (SE)			*Adjusted mean difference (95% CI)*			*Adjusted mean difference (95% CI)*
ΔCDAI	-3.1 (0.7)	-2.1 (0.7)	1.0 (-0.9, 2.8)	-4.7 (0.5)	-3.9 (0.5)	0.7 (-0.5, 2.0)
HAQ	1.1 (0.0)	1.0 (0.0)	-0.0 (-0.1, 0.1)	1.0 (0.0)	1.0 (0.0)	0.0 (-0.1, 0.1)
Patient pain (0–100 mm VAS)	46.3 (1.4)	45.2 (1.5)	-1.1 (-5.1, 3.0)	43.1 (1.1)	42.5 (1.1)	-0.6 (-3.6, 2.4)
Patient fatigue (0–100 mm VAS)	48.2 (1.4)	49.2 (1.4)	1.1 (-2.9, 5.0)	46.7 (1.1)	46.1 (1.1)	-0.5 (-3.5, 2.5)
Morning stiffness (hours)	1.6 (0.2)	1.4 (0.2)	-0.2 (-0.7, 0.3)	1.5 (0.1)	1.5 (0.1)	-0.1 (-0.4, 0.3)

	**Tofacitinib monotherapy (*N*=246)**	**TNFi combination therapy (*N*=246)**	**Comparison[j] (TNFi combination therapy vs tofacitinib monotherapy)**	**Tofacitinib combination therapy (*N*=344)**	**TNFi combination therapy (*N*=344)**	**Comparison[k] (TNFi combination therapy vs tofacitinib combination therapy)**
**All initiators**						
n/*N*, predicted probabilities, % (SE)			*Adjusted OR (95% CI)*			*Adjusted OR (95% CI)*
CDAI-LDA (CDAI ≤10)[d]	40/160, 25.4 (3.5)	52/165, 31.1 (3.6)	1.33 (0.81, 2.16)	57/234, 24.2 (2.8)	67/240, 28.1 (2.9)	1.23 (0.81, 1.87)
CDAI remission (≤2.8)[e]	20/230, 8.8 (1.9)	30/236, 12.5 (2.1)	1.49 (0.82, 2.71)	26/319, 8.1 (1.5)	23/317, 7.3 (1.5)	0.90 (0.50, 1.62)
mACR20[f]	42/244, 16.4 (2.3)	33/244, 14.3 (2.2)	0.84 (0.50, 1.40)	65/343, 18.8 (2.1)	57/341, 16.9 (2.0)	0.88 (0.59, 1.30)
mACR50[f]	21/244, 9.0 (1.9)	19/244, 8.6 (1.9)	0.95 (0.49, 1.83)	30/343, 8.7 (1.5)	32/341, 9.4 (1.6)	1.10 (0.65, 1.86)
mACR70[f]	12/244, 5.1 (1.4)	9/244, 4.1 (1.3)	0.80 (0.33, 1.95)	10/343, 2.9 (0.9)	14/341, 4.1 (1.1)	1.43 (0.62, 3.28)
HAQ MCID[g]	62/199, 30.3 (3.2)	62/201, 31.7 (3.3)	1.07 (0.69, 1.64)	94/290, 32.3 (2.7)	86/281, 30.7 (2.7)	0.93 (0.65, 1.32)
Mild pain (VAS; ≤20 mm)[h]	28/186, 15.1 (2.6)	25/191, 13.0 (2.4)	0.84 (0.47, 1.51)	39/265, 14.7 (2.2)	36/265, 13.6 (2.1)	0.91 (0.56, 1.50)
DAS28-4(ESR) LDA/remission (≤3.2)[i]	12/95, 13.0 (3.5)	17/88, 20.5 (4.4)	1.72 (0.76, 3.87)	24/151, 15.3 (2.9)	24/143, 17.5 (3.2)	1.18 (0.63, 2.23)
Adjusted mean (SE)			*Adjusted mean difference (95% CI)*			*Adjusted mean difference (95% CI)*
ΔCDAI	-3.0 (0.6)	-4.7 (0.6)	-1.7 (-3.5, 0.1)	-3.3 (0.6)	-4.3 (0.6)	-1.0 (-2.6, 0.6)
HAQ	1.0 (0.0)	1.0 (0.0)	-0.0 (-0.1, 0.1)	1.0 (0.0)	1.0 (0.0)	0.0 (-0.1, 0.1)
Patient pain (0–100 mm VAS)	46.4 (1.6)	41.5 (1.6)	-4.8 (-9.2, -0.5)	43.5 (1.3)	44.3 (1.3)	0.8 (-3.0, 4.5)
Patient fatigue (0–100 mm VAS)	49.5 (1.5)	46.4 (1.5)	-3.2 (-7.4, 1.1)	46.7 (1.3)	47.7 (1.3)	1.0 (-2.7, 4.7)
Morning stiffness (hours)	1.6 (0.2)	1.4 (0.2)	-0.2 (-0.7, 0.3)	1.4 (0.2)	1.6 (0.2)	0.2 (-0.3, 0.6)

n/*N* denotes unadjusted response rates

Predicted probabilities and ORs (binary outcomes), and mean score and mean differences (continuous outcomes) are based on adjusted outcomes following propensity score matching

[a]12-month visit occurred 10–14 months after the index date

[b]Covariates that remained imbalanced after propensity score matching and were included in the final model for the comparison of tofacitinib monotherapy versus tofacitinib

combination therapy were baseline value and work status, insurance, and history of cancer

[c]Covariates that remained imbalanced after propensity score matching and were included in the final model for the comparison of TNFi monotherapy versus TNFi combination therapy were baseline value and age, BMI, history of diabetes, and CDAI

[d]Calculated among those patients with CDAI >10 at baseline

[e]Calculated among those patients with CDAI >2.8 at baseline

[f]20%, 50%, or 70% improvement in the mACR response criteria

[g]MCID was a decrease of ≥0.22 from baseline; calculated among those patients with HAQ >0.22 at baseline

[h]Calculated among those patients with pain >20 mm VAS at baseline

[i]Calculated among those patients with DAS28-4(ESR) >3.2 at baseline

[j]Covariates that remained imbalanced after propensity score matching and were included in the final model for the comparison of tofacitinib monotherapy versus TNFi combination therapy were baseline value and history of cancer and swollen joint count

[k]Covariates that remained imbalanced after propensity score matching and were included in the final model for the comparison of tofacitinib combination therapy versus TNFi combination therapy were baseline value and age, insurance, duration of RA, and methotrexate use

Δ change from baseline, *BMI* body mass index, *CDAI* Clinical Disease Activity Index, *CI* confidence interval, *DAS28-4(ESR)* Disease Activity Score in 28 joints, erythrocyte sedimentation rate, *HAQ* Health Assessment Questionnaire, *LDA* low disease activity, *mACR* modified American College of Rheumatology, *MCID* minimum clinically important difference, *N* total number of patients, *n* number of patients with outcome, *OR* odds ratio, *RA* rheumatoid arthritis, *REF* reference treatment group, *SE* standard error, *TNFi* tumor necrosis factor inhibitor, *VAS* Visual Analog Scale

Table S5 Primary and secondary effectiveness outcomes by treatment regimen at month 6 in those with a 6-month visit[a] (propensity score matched population)

	**Tofacitinib monotherapy (*N*=345)**	**Tofacitinib combination therapy (*N*=345)**	**Comparison[b] (Tofacitinib combination therapy vs tofacitinib monotherapy)**	**TNFi monotherapy (*N*=670)**	**TNFi combination therapy (*N*=670)**	**Comparison[c] (TNFi combination therapy vs TNFi monotherapy)**
**All initiators**						
n/*N*, predicted probabilities, % (SE)			*Adjusted ORs (95% CI)*			*Adjusted ORs (95% CI)*
CDAI-LDA (CDAI ≤10)[d]	66/228, 27.0 (2.8)	48/231, 21.7 (2.7)	0.72 (0.44, 1.16)	166/469, 35.7 (2.2)	154/438, 34.9 (2.3)	0.96 (0.73, 1.27)
CDAI remission (≤2.8)[e]	29/317, 7.9 (1.5)	23/311, 8.3 (1.6)	1.06 (0.57, 1.99)	69/606, 11.5 (1.3)	78/604, 12.7 (1.3)	1.12 (0.79, 1.58)
mACR20[f]	63/333, 18.9 (2.2)	56/328, 17.1 (2.1)	0.88 (0.59, 1.34)	137/666, 20.8 (1.6)	151/663, 22.6 (1.6)	1.11 (0.86, 1.45)
mACR50[f]	38/333, 11.4 (1.8)	25/328, 8.0 (1.5)	0.67 (0.39, 1.15)	90/666, 13.7 (1.3)	85/663, 12.7 (1.3)	0.92 (0.67, 1.26)
mACR70[f]	15/333, 4.4 (1.2)	10/328, 3.5 (1.1)	0.79 (0.34, 1.83)	47/666, 7.2 (1.0)	35/663, 5.1 (0.8)	0.69 (0.44, 1.09)
HAQ MCID[g]	94/269, 35.2 (2.9)	82/278, 29.4 (2.7)	0.76 (0.53, 1.11)	202/543, 37.6 (2.1)	197/539, 36.2 (2.1)	0.94 (0.73, 1.21)
Mild pain (VAS; ≤20 mm)[h]	43/261, 15.7 (2.2)	42/265, 16.2 (2.3)	1.04 (0.62, 1.73)	107/524, 20.5 (1.8)	96/520, 18.4 (1.7)	0.87 (0.64, 1.19)
DAS28-4(ESR) LDA/remission (≤3.2)[i]	24/131, 17.1 (3.3)	20/149, 14.7 (2.9)	0.82 (0.41, 1.67)	62/255, 24.1 (2.7)	56/236, 24.0 (2.8)	1.00 (0.66, 1.52)
Adjusted mean (SE)			*Adjusted mean difference (95% CI)*			*Adjusted mean difference (95% CI)*
ΔCDAI	-3.2 (0.58)	-2.8 (0.59)	0.4 (-1.2, 2.0)	-4.1 (0.40)	-4.7 (0.40)	-0.6 (-1.7, 0.6)
HAQ	1.1 (0.0)	1.1 (0.0)	-0.0 (-0.1, 0.1)	0.9 (0.0)	0.9 (0.0)	-0.0 (-0.1, 0.0)
Patient pain (0–100 mm VAS)	44.7 (1.26)	45.6 (1.28)	0.8 (-2.7, 4.4)	42.9 (0.97)	41.6 (0.97)	-1.3 (-3.9, 1.5)
Patient fatigue (0–100 mm VAS)	48.3 (1.17)	46.6 (1.19)	-1.7 (-5.0, 1.6)	46.1 (0.93)	45.0 (0.93)	-1.2 (-3.8, 1.5)
Morning stiffness (hours)	1.4 (0.15)	1.46 (0.15)	0.0 (-0.4, 0.4)	1.6 (0.11)	1.4 (0.11)	-0.2 (-0.5, 0.1)

	**Tofacitinib monotherapy (*N*=343)**	**TNFi combination therapy (*N*=343)**	**Comparison[j] (TNFi combination therapy vs tofacitinib monotherapy)**	**Tofacitinib combination therapy (*N*=443)**	**TNFi combination therapy (*N*=443)**	**Comparison[k] (TNFi combination therapy vs tofacitinib combination therapy)**
**All initiators**						
n/*N*, predicted probabilities, % (SE)			*Adjusted ORs (95% CI)*			*Adjusted ORs (95% CI)*
CDAI-LDA (CDAI ≤10)[d]	59/223, 26.4 (3.0)	68/222, 30.3 (3.0)	1.22 (0.79, 1.88)	82/310, 25.8 (2.4)	82/301, 28.3 (2.6)	1.14 (0.79, 1.67)
CDAI remission (≤2.8)[e]	28/315, 8.9 (1.6)	36/316, 11.5 (1.8)	1.33 (0.77, 2.29)	34/414, 7.9 (1.3)	41/412, 10.2 (1.5)	1.35 (0.81, 2.24)
mACR20[f]	66/338, 18.3 (2.0)	66/340, 20.6 (2.2)	1.18 (0.78, 1.77)	91/442, 20.1 (1.9)	91/439, 21.2 (2.0)	1.07 (0.76, 1.49)
mACR50[f]	36/338, 10.1 (1.6)	37/340, 11.6 (1.8)	1.17 (0.71, 1.95)	47/442, 10.4 (1.5)	39/439, 9.1 (1.4)	0.87 (0.55, 1.37)
mACR70[f]	13/338, 3.6 (1.0)	12/340, 3.7 (1.1)	1.04 (0.45, 2.39)	24/442, 5.5 (1.1)	16/439, 3.5 (0.9)	0.62 (0.32, 1.20)
HAQ MCID[g]	97/269, 35.8 (2.9)	95/286, 33.9 (2.8)	0.92 (0.64, 1.32)	121/379, 31.7 (2.4)	131/381, 34.8 (2.4)	1.16 (0.84, 1.59)
Mild pain (VAS; ≤20 mm)[h]	44/256, 16.6 (2.3)	40/258, 15.1 (2.2)	0.89 (0.55, 1.46)	62/348, 17.6 (2.0)	56/348, 16.4 (2.0)	0.91 (0.60, 1.38)
DAS28-4(ESR) LDA/remission (≤3.2)[i]	23/129, 18.0 (3.5)	32/129, 24.8 (3.8)	1.54 (0.80, 2.94)	36/201, 17.4 (2.6)	34/167, 21.1 (3.2)	1.29 (0.73, 2.27)
Adjusted mean (SE)			*Adjusted mean difference (95% CI)*			*Adjusted mean difference (95% CI)*
ΔCDAI	-3.0 (0.56)	-4.1 (0.55)	-1.1 (-2.7, 0.5)	-3.8 (0.49)	-4.7 (0.49)	-0.9 (-2.3, 0.4)
HAQ	1.0 (0.0)	1.0 (0.0)	0.0 (-0.1, 0.1)	1.1 (0.0)	1.1 (0.0)	-0.0 (-0.1, 0.4)
Patient pain (0–100 mm VAS)	43.7 (1.33)	43.1 (1.33)	-0.7 (-4.4, 3.1)	44.3 (1.15)	43.9 (1.14)	-0.4 (-3.6, 2.8)
Patient fatigue (0–100 mm VAS)	47.4 (1.27)	48.6 (1.27)	1.2 (-2.4, 4.7)	45.5 (1.08)	47.9 (1.08)	2.4 (-0.7, 5.4)
Morning stiffness (hours)	1.3 (0.12)	1.3 (0.12)	-0.0 (-0.3, 0.3)	1.5 (0.12)	1.4 (0.12)	-0.0 (-0.4, 0.3)

n/*N* denotes unadjusted response rates

Predicted probabilities and ORs (binary outcomes), and mean score and mean differences (continuous outcomes) are based on adjusted outcomes following propensity score matching

[a]6-month visit occurred 4–8 months after the index date

[b]Covariates that remained imbalanced after propensity score matching and were included in the final model for the comparison of tofacitinib monotherapy versus tofacitinib

combination therapy were baseline value and race, work status, insurance, and HAQ

[c]Covariates that remained imbalanced after propensity score matching and were included in the final model for the comparison of TNFi monotherapy versus TNFi combination therapy were baseline value and age, and race

[d]Calculated among those patients with CDAI >10 at baseline

[e]Calculated among those patients with CDAI >2.8 at baseline

[f]20%, 50%, or 70% improvement in the mACR response criteria

[g]MCID was a decrease of ≥0.22 from baseline; calculated among those patients with HAQ >0.22 at baseline

[h]Calculated among those patients with pain >20 mm VAS at baseline

[i]Calculated among those patients with DAS28-4(ESR) >3.2 at baseline

[j]Covariates that remained imbalanced after propensity score matching and were included in the final model for the comparison of tofacitinib monotherapy versus TNFi combination therapy were baseline value and race, BMI, history of diabetes, history of non-TNFi use, and tender joint count

[k]Covariates that remained imbalanced after propensity score matching and were included in the final model for the comparison of tofacitinib combination therapy versus TNFi combination therapy were baseline value and age, race, work status, insurance, history of cancer, duration of RA, HAQ, and methotrexate use

Δ change from baseline, *BMI* body mass index, *CDAI* Clinical Disease Activity Index, *CI* confidence interval, *DAS28-4(ESR)* Disease Activity Score in 28 joints, erythrocyte sedimentation rate, *HAQ* Health Assessment Questionnaire, *LDA* low disease activity, *mACR* modified American College of Rheumatology, *MCID* minimum clinically important difference, *N* total number of patients, *n* number of patients with outcome, *OR* odds ratio, *RA* rheumatoid arthritis, *REF* reference treatment group, *SE* standard error, *TNFi* tumor necrosis factor inhibitor, *VAS* Visual Analog Scale

Table S6 Secondary effectiveness outcomes by tofacitinib line of therapy at month 12 in those with a 12-month visit[a] (unmatched population)

	**2nd-line tofacitinib (*N*=135)**	**3rd-line tofacitinib (*N*=157)**	**≥4th-line tofacitinib (*N*=513)**
Response rates, *n* (%) [SE]			
CDAI remission (≤2.8)[b]	25 (19.8) [0.0]	11 (7.9) [0.0]	31 (6.5) [0.0]
mACR20[c]	30 (23.4) [0.0]	30 (19.4) [0.0]	79 (15.7) [0.0]
mACR50[c]	20 (15.6) [0.0]	11 (7.1) [0.0]	34 (6.8) [0.0]
mACR70[c]	6 (4.7) [0.0]	5 (3.2) [0.0]	17 (3.4) [0.0]
HAQ MCID[d]	38 (49.4) [0.1]	43 (53.8) [0.1]	127 (57.2) [0.0]
Mild pain (VAS; ≤20 mm)[e]	25 (28.4) [0.1]	18 (15.9) [0.0]	50 (12.1) [0.0]
DAS28-4(ESR) LDA/remission (≤3.2)[f]	17 (27.4) [0.1]	11 (16.9) [0.1]	20 (9.9) [0.0]
Mean (SD)			
ΔCDAI	-3.8 (12.2)	-3.8 (11.5)	-2.8 (13.8)
HAQ	0.7 (0.7)	0.9 (0.8)	1.1 (0.7)
Patient pain (0–100 mm VAS)	31.7 (27.4)	40.0 (27.4)	49.0 (28.5)
Patient fatigue (0–100 mm VAS)	35.6 (29.1)	46.0 (31.2)	51.0 (30.0)
Morning stiffness (hours)	0.9 (2.3)	1.3 (2.5)	1.6 (3.2)

N for each specific outcome may vary

Data shown are not propensity score matched. Response rates and mean scores are based on unadjusted outcomes

[a]12-month visit occurred 10–14 months after the index date

[b]Calculated among those patients with CDAI >2.8 at baseline

[c]20%, 50%, or 70% improvement in the mACR response criteria

[d]MCID was a decrease of ≥0.22 from baseline; calculated among those patients with HAQ >0.22 at baseline

[e]Calculated among those patients with pain >20 mm VAS at baseline

[f]Calculated among those patients with DAS28-4(ESR) >3.2 at baseline

Δ change from baseline, *CDAI* Clinical Disease Activity Index, *DAS28-4(ESR)* Disease Activity Score in 28 joints, erythrocyte sedimentation rate, *HAQ* Health Assessment Questionnaire, *LDA* low disease activity, *mACR* modified American College of Rheumatology, *MCID* minimum clinically important difference, *N* total number of patients, *n* number of patients with outcome, *SD* standard deviation, *SE* standard error, *VAS* Visual Analog Scale

Table S7 Comparisons of secondary effectiveness outcomes across different lines of tofacitinib therapy at month 12 in those with a 12-month visit[a] (unmatched population)

	**Comparison (3rd-line vs 2nd-line tofacitinib)**	**Comparison (3rd-line vs ≥4th-line tofacitinib)**
OR (95% CI)		
CDAI remission (≤2.8)[b]	0.37 (0.16, 0.83)	0.94 (0.44, 2.03)
mACR20[c]	0.82 (0.44, 1.53)	1.49 (0.90, 2.47)
mACR50[c]	0.45 (0.20, 1.02)	1.19 (0.57, 2.48)
mACR70[c]	0.81 (0.23, 2.80)	0.97 (0.34, 2.78)
HAQ MCID[d]	1.25 (0.61, 2.54)	0.85 (0.48, 1.52)
Mild pain (VAS; ≤20 mm)[e]	0.50 (0.25, 1.04)	1.14 (0.62, 2.11)
DAS28-4(ESR) LDA/remission (≤3.2)[f]	0.48 (0.18, 1.28)	1.26 (0.51, 3.12)
Mean difference (95% CI)		
ΔCDAI	0.2 (-2.4, 2.8)	-2.6 (-4.6, -0.5)
HAQ	0.2 (0.0, 0.3)	-0.2 (-0.3, -0.0)
Patient pain (0–100 mm VAS)	8.8 (2.4, 15.1)	-7.0 (-12.0, -2.0)
Patient fatigue (0–100 mm VAS)	11.5 (4.7, 18.3)	-2.3 (-7.6, 3.0)
Morning stiffness (hours)	0.5 (-0.2, 1.2)	-0.2 (-0.7, 0.4)

Data shown are not propensity score matched. ORs (95% CI) and mean differences are based on models adjusted for tofacitinib exposure, age, duration of RA, race, sex, and baseline CDAI

[a]12-month visit occurred 10–14 months after the index date

[b]Calculated among those patients with CDAI >2.8 at baseline

[c]20%, 50%, or 70% improvement in the mACR response criteria

[d]MCID was a decrease of ≥0.22 from baseline; calculated among those patients with HAQ >0.22 at baseline

[e]Calculated among those patients with pain >20 mm VAS at baseline

[f]Calculated among those patients with DAS28-4(ESR) >3.2 at baseline

Δ change from baseline, *CDAI* Clinical Disease Activity Index, *CI* confidence interval, *DAS28-4(ESR)* Disease Activity Score in 28 joints, erythrocyte sedimentation rate, *HAQ* Health Assessment Questionnaire, *LDA* low disease activity, *mACR* modified American College of Rheumatology, *MCID* minimum clinically important difference, *OR* odds ratio, *RA* rheumatoid arthritis, *VAS* Visual Analog Scale

Table S8 Primary and secondary effectiveness outcomes by tofacitinib line of therapy at month 6 in those with a 6-month visit[a] (unmatched population)

	**2nd-line tofacitinib (*N*=171)**	**3rd-line tofacitinib (*N*=193)**	**≥4th-line tofacitinib (*N*=625)**
Response rates, *n* (%) [SE]			
CDAI-LDA (CDAI ≤10)[b]	40 (36.4) [0.1]	37 (30.3) [0.0]	99 (22.5) [0.0]
CDAI Remission (≤2.8)[c]	28 (17.7) [0.0]	14 (8.0) [0.0]	38 (6.5) [0.0]
mACR20[d]	37 (22.6) [0.0]	30 (16.1) [0.0]	113 (18.8) [0.0]
mACR50[d]	19 (11.6) [0.0]	21 (11.3) [0.0]	55 (9.2) [0.0]
mACR70[d]	12 (7.3) [0.0]	9 (4.8) [0.0]	23 (3.8) [0.0]
HAQ MCID[e]	44 (36.7) [0.0]	46 (30.3) [0.0]	167 (32.4) [0.0]
Mild pain (VAS; ≤20 mm)[f]	29 (25.2) [0.0]	30 (20.7) [0.0]	71 (14.2) [0.0]
DAS28-4(ESR) LDA/remission (≤3.2)[g]	17 (21.5) [0.1]	18 (21.4) [0.0]	38 (15.3) [0.0]
Mean (SD)			
ΔCDAI	-5.2 (11.6)	-4.0 (11.4)	-3.1 (13.4)
HAQ	0.8 (0.7)	1.0 (0.7)	1.1 (0.7)
Patient pain (0–100 mm VAS)	33.2 (28.6)	40.8 (29.6)	47.3 (28.5)
Patient fatigue (0–100 mm VAS)	34.8 (28.4)	42.1 (29.8)	50.9 (28.7)
Morning stiffness (hours)	1.2 (3.1)	1.0 (1.2)	1.7 (3.4)

N for each specific outcome may vary

Data shown are not propensity score matched. Response rates and mean scores are based on unadjusted outcomes

[a]6-month visit occurred 4–8 months after the index date

[b]Calculated among those patients with CDAI >10 at baseline

[c]Calculated among those patients with CDAI >2.8 at baseline

[d]20%, 50%, or 70% improvement in the mACR response criteria

[e]MCID was a decrease of ≥0.22 from baseline; calculated among those patients with HAQ >0.22 at baseline

[f]Calculated among those patients with pain >20 mm VAS at baseline

[g]Calculated among those patients with DAS28-4(ESR) >3.2 at baseline

Δ change from baseline, *CDAI* Clinical Disease Activity Index, *DAS28-4(ESR)* Disease Activity Score in 28 joints, erythrocyte sedimentation rate, *HAQ* Health Assessment Questionnaire, *LDA* low disease activity, *mACR* modified American College of Rheumatology, *MCID* minimum clinically important difference, *N* total number of patients, *n* number of patients with outcome, *SD* standard deviation, *SE* standard error, *VAS* Visual Analog Scale

Table S9 Comparisons of secondary effectiveness outcomes across different lines of tofacitinib therapy at month 6 in those with a 6-month visit[a] (unmatched population)

	**Comparison (3rd-line vs 2nd-line tofacitinib)**	**Comparison (3rd-line vs ≥4th-line tofacitinib)**
OR (95% CI)		
CDAI-LDA (CDAI ≤10)[b]	0.71 (0.39, 1.27)	1.35 (0.84, 2.16)
CDAI remission (≤2.8)[c]	0.43 (0.21, 0.88)	1.13 (0.58, 2.17)
mACR20[d]	0.71 (0.40, 1.27)	1.01 (0.63, 1.63)
mACR50[d]	1.14 (0.57, 2.26)	1.36 (0.79, 2.36)
mACR70[d]	0.77 (0.30, 1.96)	1.40 (0.63, 3.13)
HAQ MCID[e]	0.74 (0.44, 1.25)	0.93 (0.62, 1.40)
Mild pain (VAS; ≤20 mm)[f]	0.76 (0.42, 1.37)	1.52 (0.94, 2.46)
DAS28-4(ESR) LDA/remission (≤3.2)[g]	1.11 (0.49, 2.52)	1.27 (0.66, 2.46)
Mean difference (95% CI)		
ΔCDAI	0.2 (-2.0, 2.4)	-2.4 (-4.1, -0.7)
HAQ	0.1 (-0.0, 0.2)	-0.1 (-0.2, -0.0)
Patient pain (0–100 mm VAS)	7.6 (1.9, 13.4)	-4.7 (-9.3, -0.2)
Patient fatigue (0–100 mm VAS)	7.0 (1.2, 12.8)	-6.9 (11.5, -2.4)
Morning stiffness (hours)	-0.1 (-0.8, 0.5)	-0.6 (-1.1, -0.1)

Data shown are not propensity score matched. ORs (95% CIs) and mean differences are based on models adjusted for tofacitinib exposure, age, duration of RA, race, sex, and baseline CDAI

[a]6-month visit occurred 4–8 months after the index date

[b]Calculated among those patients with CDAI >10 at baseline

[c]Calculated among those patients with CDAI >2.8 at baseline

[d]20%, 50%, or 70% improvement in the mACR response criteria

[e]MCID was a decrease of ≥0.22 from baseline; calculated among those patients with HAQ >0.22 at baseline

[f]Calculated among those patients with pain >20 mm VAS at baseline

[g]Calculated among those patients with DAS28-4(ESR) >3.2 at baseline

Δ change from baseline, *CDAI* Clinical Disease Activity Index, *CI* confidence interval, *DAS28-4(ESR)* Disease Activity Score in 28 joints, erythrocyte sedimentation rate, *HAQ* Health Assessment Questionnaire, *LDA* low disease activity, *mACR* modified American College of Rheumatology, *MCID* minimum clinically important difference, *OR* odds ratio, *RA* rheumatoid arthritis, *VAS* Visual Analog Scale
